# Diversification across an altitudinal gradient in the Tiny Greenbul (*Phyllastrephus debilis*) from the Eastern Arc Mountains of Africa

**DOI:** 10.1186/1471-2148-11-117

**Published:** 2011-05-03

**Authors:** Jérôme Fuchs, Jon Fjeldså, Rauri CK Bowie

**Affiliations:** 1 Museum of Vertebrate Zoology and Department of Integrative Biology, 3101 Valley Life Science Building, University of California, Berkeley, CA, 94720-3160, USA; 2 DST/NRF Centre of Excellence at the Percy FitzPatrick Institute, University of Cape Town, Rondebosch 7701, South Africa; 3 Centre of Macroecology, Evolution and Climate, Zoological Museum, University of Copenhagen, Universitetsparken 15, DK-2100 Copenhagen, Denmark; 4 California Academy of Sciences, 55 Music Concourse Drive, San Francisco, CA, 94118, USA

## Abstract

**Background:**

The Eastern Arc Mountains of Africa have become one of the focal systems with which to explore the patterns and mechanisms of diversification among montane species and populations. One unresolved question is the extent to which populations inhabiting montane forest interact with those of adjacent lowland forest abutting the coast of eastern Africa. The Tiny Greenbul (*Phyllastephus debilis*) represents the only described bird species within the Eastern Arc/coastal forest mosaic, which is polytypic across an altitudinal gradient: the subspecies *albigula *(green head) is distributed in the montane Usambara and Nguru Mountains whereas the subspecies *rabai *(grey head) is found in Tanzanian lowland and foothill forest. Using a combination of morphological and genetic data, we aim to establish if the pattern of morphological differentiation in the Tiny Greenbul (*Phyllastrephus debilis*) is the result of disruptive selection along an altitudinal gradient or a consequence of secondary contact following population expansion of two differentiated lineages.

**Results:**

We found significant biometric differences between the lowland (*rabai*) and montane (*albigula*) populations in Tanzania. The differences in shape are coupled with discrete differences in the coloration of the underparts. Using multi-locus data gathered from 124 individuals, we show that lowland and montane birds form two distinct genetic lineages. The divergence between the two forms occurred between 2.4 and 3.1 Myrs ago.

Our coalescent analyses suggest that limited gene flow, mostly from the subspecies *rabai *to *albigula*, is taking place at three mid-altitude localities, where lowland and montane rainforest directly abut. The extent of this introgression appears to be limited and is likely a consequence of the recent expansion of *rabai *further inland.

**Conclusion:**

The clear altitudinal segregation in morphology found within the Tiny Greenbul is the result of secondary contact of two highly differentiated lineages rather than disruptive selection in plumage pattern across an altitudinal gradient. Based on our results, we recommend *albigula *be elevated to species rank.

## Background

**Figure 1:**
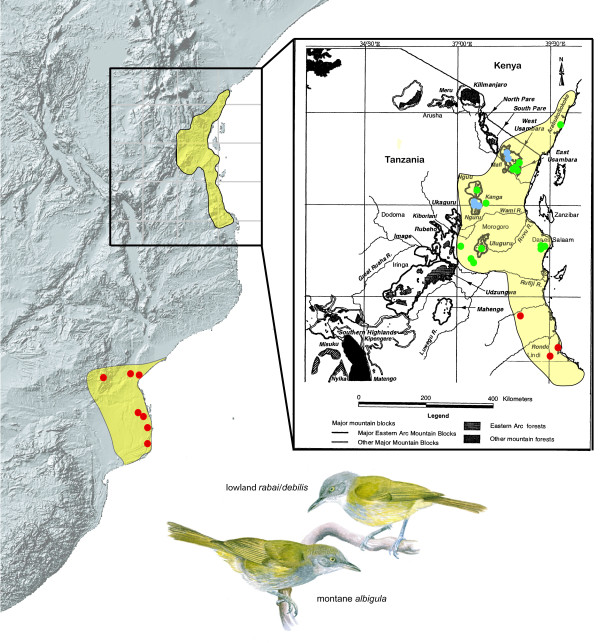
**Distribution of *Phyllastrephus debilis*; dots represent our sampling localities (blue: *albigula*; green, *rabai*; red, *debilis*)**. The painting depicts the typical plumage of *P*. *d. albigula *and *P. d. rabai/debilis*, respectively.

The Eastern Arc and coastal forests of East Africa have been identified as a biodiversity hotspot: an area featuring exceptional concentrations of endemic species and experiencing severe habitat loss [1]. The extensive botanical endemism and richness is paralleled in several animal groups [2] and has been especially well documented for birds [3,4]. The Eastern Arc Mountains consists of 13 sky islands, which form a chain (ca. 650 km long) of uplifted fault blocks extending from the Taita Hills in the northeast to the Udzungwa Mountains in the southwest (Figure 1). These mountains are under direct climatic influence from the Indian Ocean and reach an altitude of 2635 m (Uluguru Mts) [5]. Typically montane forest occurs above 800 m, but there is no sharp change in turnover of arboreal species, and the composition of the botanical communities depend on the inclination of the terrain, rainfall, distance from the coast, height and incidence of cloud cover [2,6,7]. At lower elevations (500-800 m), the eastern slopes typically sharply grade into savanna or lowland rainforest characteristic of the coastal forests of the littoral plain of Africa that runs from Somalia to Mozambique. The drier western and northwestern slopes typically support deciduous woodland at lower elevations.

Extensive field research within the Eastern Arc has taken place over the past 15 years which has led to the description of several new species and the development of novel hypotheses concerning patterns of differentiation among species and populations distributed in East Africa [4,8,9]. Despite this accumulating body of knowledge, it still remains to be determined to what extent populations inhabiting montane forest interact with populations distributed in adjacent foothills and along a narrow ribbon of lowland forest abutting the coast of eastern Africa [e.g. [10,11]].

The Tiny Greenbul (*Phyllastephus debilis*) represents the only described bird species within the Eastern Arc/coastal forest mosaic, which is polytypic across an altitudinal gradient (clinal variation is also well documented for some frogs, [12]). The more common pattern is for polytypic taxa to be distributed as a series of allopatric populations restricted to specific sky islands. Three subspecies are currently recognized [13]: *albigula *in the Usambara Mountains (Mts) as well as in the Nguru Mts 130 km further inland (Tanzania, Figure 1), *debilis *in the coastal forest zone of southeastern Tanzania, Mozambique and eastern Zimbabwe, and *rabai *which extends from near Dares Salaam (Tanzania) north along the coast into Kenya, and inland along riverine corridors to the foothill forests od the the Uluguru, Nguu and Nguru Mts (Figure 1). Subspecies *albigula *is the most distinctive form, the top of the head being green and not grey as in *debilis *and *rabai*. Unlike the other forms *albigula *is only found above 600 m, mainly in mature forest, and extends up to 1600 m in the Nguru Mts [14] and to 2150 m in the West Usambara Mts. This suggests that the green-headed montane birds represent two separate populations (Usambara Mts, Nguru Mts; Figure 1) nested inside the geographical range of a more widespread lowland, grey-headed, form. We therefore have to consider the possibility that two ecologically segregated species are involved. However, the existence of some greenish feather edges on the nape and crown of some lowland birds could lead to the alternate interpretation that although gene flow occurs, plumage is under directional selection, with greener plumage being favored in the wetter high-altitude habitats.

Whether disruptive selection across an altitudinal gradient can lead to parapatric speciation is hotly debated, as it requires natural selection to be sufficiently strong to counteract the effects of gene flow [e.g. [15-17]]. Although several studies have provided evidence of the importance of natural selection for the formation of new species even when gene flow occurs [18], research that connects presumably adaptive variation in traits (plumage in our case) with assessments of the phylogenetic relationships among populations/taxa, extent of genetic variation and gene flow are relatively few [19]. Thus, a careful analysis of morphological variation and phylogeographic structure within the Tiny Greenbul is likely to provide further insight to this debate. We contend that if populations from the same habitat (lowland or montane) are genetically and morphologically more closely related to each other than to population from the other habitat, we would expect historical isolation and secondary contact between the lowland and montane populations to be the prevailing hypothesis for the current contact zone. In contrast, if disruptive selection has played a major role in driving phenotypic divergence across the altitudinal gradient, then the two populations of the montane form (Nguru and Usambara Mts) should be phenotypically more similar despite being genetically closer to their nearest geographically lowland forest population. In this paper we make use of a multi-locus approach to test the above hypotheses.

## Results

### Morphological variation

Birds from the lowland coastal forests of East Africa (n = 49), subspecies *rabai*, are generally small (Male: weight 14.2 ± 0.57 g, wing 68.3 ± 2.4 mm, bill 13.7 ± 0.82 mm, tail 64.0 ± 2.7 mm, tarsus 17.1 ± 0.94; Female: weight 14.4 ± 0.48 g, wing 66.1 ± 3.1 mm, bill 13.5 ± 0.74 mm, tail 61.1 ± 3.7 mm, tarsus 17.7 ± 0.69) with a mouse grey crown, pale grey underparts with bright yellow streaks (Picric Yellow lateral feather edges) contrasting with a very pale grey throat and extensive pale yellow belly. This description also applies to birds from Mt. Kanga (n = 6), the Nguu Mts (n = 2) and the Uluguru foothills (n = 10; see Figure 1).

Specimens of the montane subspecies *albigula *from the Usambara (n = 6) and Nguru Mts (n = 11) are generally large (Male: weight 17.8 ± 1.4 g, wing 73.5 ± 3.4 mm, bill 14.8 ± 0.57 mm, tail 69.9 ± 2.1 mm, tarsus 19.0 ± 0.43; Female: weight 15.3 ± 0.84 g, wing 70.7 ± 1.8 mm, bill 14.0 ± 0.86 mm, tail 66.8 ± 3.3 mm, tarsus 18.0 ± 0.47). Birds of both sexes from the two montane populations of *albigula *do not differ significantly from each other in any measured morphometric trait (t-test: Male *P *= 0.15-0.61, Female *P *= 0.56-0.97), but do differ from lowland birds, with lowland birds being significantly smaller (*rabai *vs. *albigula*, t-test: Male all P < 0.01, Female weight P = 0.94, wing P = 0.23, bill P = 0.29, tail P = 0.04, tarsus P = 0.47).

The first two principal components (PCs) had eigenvalues greater than one and accounted for 77.2% of the total variation (Factor 1 45.5%, Factor 2 31.7%). Principal component loadings on PC1 were positive and strongly correlated with weight. Loadings on PC2 were positive and strongly correlated with wing- and tail-length; bill- and tarsus-length loaded evenly on both PC1 and PC2. In agreement with the bivariate results above, the scatterplot (Additional File 1) suggests that montane populations of *debilis *(*albigula*) are separable from lowland populations (*rabai*) in morphospace, particularly along PC1 (weight and tarsus-length, i.e. indicators of size).

In plumage characters, the dark olive-green color of the back of *albigula *extends to the crown or even upper forehead. However, in some individuals the crown feathers are grey with green lateral edges, giving a streaked appearance. The birds have a distinctive dark grey breast and flanks (Gull Grey to Plumbeous Grey, grading to Deep Grape Green on the upper sides) and even the throat and chin are rather grey (Pale Gull Grey, somewhat mottled) leaving only a narrow area on the central belly white. Yellow streaks are variably developed but generally weak and there is always a bright yellow axillary patch which, together with the yellow wing-lining, stands out conspicuously. Thus, the most prominent plumage difference between the *rabai *and *albigula *specimens is in the color of the underparts rather than the crown as traditionally described.

One bird from Mt. Kanga collected at 900 m (ZMUC101292, tissue no. 132719), a locality within the range of lowland *rabai*, resembles individuals from the adjacent Nguru Mts (*albigula*) in plumage and size (Male: weight 14.5 g, wing 76 mm, tail 71 mm). The ZMUC specimen (74473) from Lindi (southern Tanzanian coast, subspecies *debilis*) we sequenced in this study resembled specimens from lowland populations further north on the Tanzanian coast (*rabai*) in both plumage and size (Male: wing 67 mm, bill 13.7 mm, tail 59 mm, tarsus 17.2 mm). Only one specimen from Zimbabwe (*debilis*) could be compared directly with Tanzanian specimens, and differs from the Lindi specimen and most of the other *rabai *specimens only in having a faint greenish tinge on the crown. The description in Keith et al. [20] suggests that plumage and size differences between *rabai *and *debilis *are slight.

In conclusion, there exist significant biometric differences between the lowland (*rabai*) and montane (*albigula*) populations in Tanzania (our sample size for *debilis *was too small to allow for a meaningful comparison among lowland taxa). The differences in shape are also coupled with discrete differences in the color of the underparts.

### Genetic analyses: geographic structure

#### Mitochondrial data

We obtained the complete ND2 sequence (1041 bp) for 110 individuals. For some museum samples, we were only able to gather partial sequences. These partial sequences were very similar to the corresponding fragments obtained from individuals collected at the same locality; however, we did not include individuals with partial sequences in our final analyses. All sequences had an open reading frame, with no insertions, deletions or unexpected stop codons.

Within *P. debilis*, 170 sites were variable, delineating 43 haplotypes (Hd = 0.948, π = 0.05776). Results of the MacDonald-Kreitman test when comparing the *P. debilis *(n = 110) sequences with two of its closest relatives indicate no consistent evidence of selection (*P. hypochloris P *= 0.18; *P. xavieri P *= 0.04), although one comparison is marginally significant. We attribute this to homoplasy as a result of the long divergence time between *P. debilis *and its closest relatives. Further, comparing the two primary clades (*albigula *versus *debilis*/*rabai *see below) with each other indicated that no selection is acting on the mtDNA locus within *P. debilis *(*P *= 0.85).

**Figure 2:**
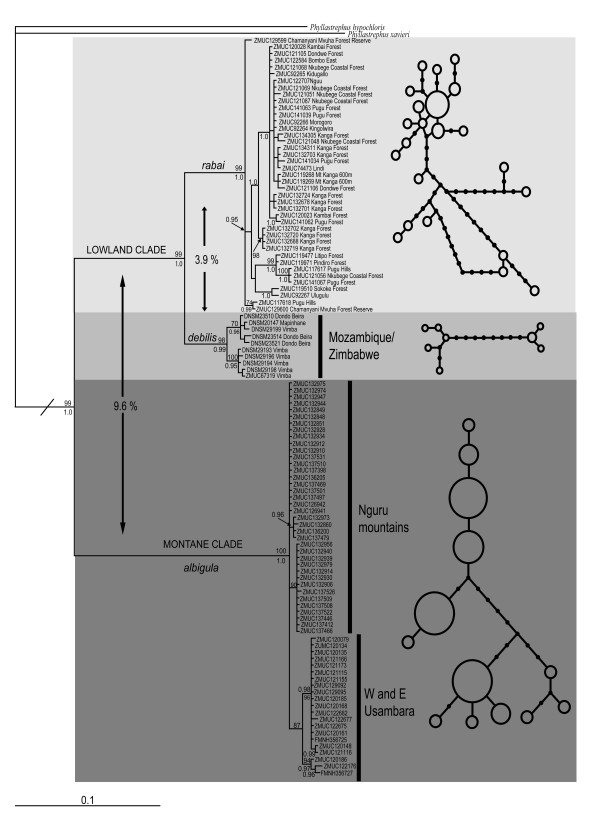
**50% majority consensus rule tree obtained from the Bayesian analyses of ND2**. Values close to nodes represent bootstrap values (above node; if > 75%) and Bayesian posterior probabilities (below node; if >0.95). Mean genetic distances among the primary groups are indicated. The haplotype networks were constructed using the statistical parsimony algorithm implemented in TCS.

Both the maximum likelihood (-ln = 3400.03) and Bayesian inference (harmonic mean -ln = 3714.18) analyses, performed using a HKY+Γ model, recovered two primary clades (net sequence divergence 9.6%); one clade includes birds collected in the lowland forests of Tanzania (*rabai*) and Mozambique/Zimbabwe (*debilis*) and the other in the montane forests of Tanzania (*albigula*) (Figure 2 and summary statistics in Table 1). Birds from the lowland forests are further subdivided into two clades, one restricted to Tanzania and one distributed in Mozambique/Zimbabwe (net sequence divergence: 3.9%). We only found three mismatches between subspecies designation and our assignment of individuals to a particular clade. The three SE Tanzanian individuals we sampled (Lindi, Litipo and Pindiro Forests, Figures 1 and 2), which according to the accepted taxonomy, should be associated with the Mozambique/Zimbabwe lowland populations (*debilis*), were placed together with the more northerly Tanzanian lowland populations (*rabai*). In all subsequent analyses, we considered the individuals sampled in Lindi, Litipo and Pindiro Forests as being part of the subspecies *rabai *and different from the subspecies *debilis*, which we considered restricted to Mozambique and Zimbabwe.

**Table 1:** Genetic diversity estimated using DNASP for each locus: Hd, haplotype diversity; S, number of segregating sites and π, nucleotide diversity.

Locus	Nbp/Nvar	Population	N_alleles_	θ (from S)	N_haplotype_	Hd	S	π	Fu's *Fs/R_2_*
**ND2**	1041/170	*debilis sensu lato*	110	0.03106	43	0.948	170	0.05776	11.200/0.1757

		*albigula *(Nguru + Usambara Mts)	59	0.00539	13	0.844	25	0.00586	1.303/0.1175

		Nguru Mts	38	0.00161	6	0.751	7	0.00215	1.120/0.0839

		Usambara Mts	21	0.00375	7	0.562	14	0.00239	-0.458/0.1553

		Lowland (*rabai *+ *debilis sensu stricto*)	51	0.02034	30	0.965	95	0.02068	-1.521//0.1103

		*rabai*	41	0.01216	22	0.948	54	0.00833	-3.976**/0.0740

		*debilis sensu stricto*	10	0.00579	8	0.956	17	0.00681	-1.104/0.1827

**FGB**	559/17	*debilis sensu lato*	184	0.00525	17	0.575	17	0.00432	-3.383*/0.0706

		*albigula *(Nguru + Usambara Mts)	118	0.00067	3	0.05	2	0.00009	-3.527*/0.0466*

		Nguru Mts	76	0.00036	2	0.052	1	0.00009	-1.248/0.0260**

		Usambara Mts	42	0.00042	2	0.048	1	0.00009	-1.149/0.1525

		Lowland (*rabai *+ *debilis sensu stricto*)	66	0.00564	15	0.777	15	0.00460	-4.127**/0.0852

		*rabai*	58	0.00425	10	0.713	11	0.00401	-1.198/0.1018

		*debilis sensu stricto*	8	0.00483	6	0.929	7	0.00530	-1.637/0.1866

**GAPDH**	328/15	*debilis sensu lato*	208	0.00776	20	0.827	15	0.00622	-7.122**/0.0676

		*albigula *(Nguru + Usambara Mts)	110	0.00464	11	0.824	8	0.00619	-1.261/0.1266

		Nguru Mts	68	0.00511	10	0.824	8	0.00683	-0.934/0.1397

		Usambara Mts	42	0.00355	6	0.661	5	0.00329	-1.016/0.1074

		Lowland (*rabai *+ *debilis sensu stricto*)	98	0.00771	16	0.556	13	0.00351	-10.918**/0.0439

		*rabai*	70	0.00762	15	0.634	12	0.00443	-8.485**/0.0596

		*debilis sensu stricto*	28	0.00157	3	0.320	2	0.00103	-0.731/0.0840

**BRM**	364/12	*debilis sensu lato*	168	0.00530	14	0.849	11	0.00768	-0.914/0.1257

		*albigula *(Nguru + Usambara Mts)	96	0.00214	4	0.639	4	0.00226	0.852/0.1100

		Nguru Mts	62	0.00264	4	0.633	4	0.00264	0.947/0.1284

		Usambara Mts	34	0.00067	2	0.428	1	0.00118	1.407/0.2139

		Lowland (*rabai *+ *debilis sensu stricto*)	72	0.00397	10	0.815	7	0.00511	-1.598/0.1328

		*rabai*	58	0.00415	9	0.759	7	0.00508	-1.221/0.1321

		*debilis sensu stricto*	14	0.00259	4	0.692	3	0.00353	0.135/0.2143

In the absence of selection, significant negative values of Fu's *Fs *and low values of *R_2 _*are indicative of population expansion (*p < 0.05, **p < 0.01). For the three nuclear loci, only alleles with phase probabilities greater than 0.75 were included in the analyses. The subspecies *rabai *includes all individuals from lowland Tanzania (including the three specimens from SE Tanzania, whereas subspecies *debilis *only includes individuals from Mozambique and Zimbabwe, see text for details).

Greater genetic diversity was observed in the Tanzanian lowland clade (*rabai*) than in the montane clade (*albigula*) (Table 1, Figure 2). The Mozambique/Zimbabwe clade is further divided into two subclades (Zimbabwe versus coastal Mozambique forests) that differ from each other by a minimum of nine substitutions. One individual, collected in Vimba (Zimbabwe, DM29199), possesses a mtDNA haplotype that is nested within the Mozambique subclade (Beira/Mapinhane). The three primary clades (montane, lowland Tanzania and Mozambique/Zimbabwe) could not be connected at the 95% level in the TCS haplotype network (Figure 2). Samples from the two isolated mountains where *albigula *occurs were segregated genetically; haplotypes from the Nguru and Usambara Mts were reciprocally monophyletic in the ML tree (bootstrap: 70% and 87%, respectively) and TCS network, but not in the 50% majority rule consensus tree resulting from the Bayesian analyses, where the Nguru haplotypes were paraphyletic with respect to the Usambara haplotypes (Figure 2).

We only found evidence for isolation by distance in the Tanzanian clade (*rabai*) (Mantel's test, r = 0.173, P = 0.019).

#### Autosomal intron data

The HKA test did not detect any evidence of selection between the two autosomal data sets (FIB vs. GAPDH: χ^2 ^= 0.429, P = 0.51), nor between the autosomal and sex-linked locus (GAPDH vs. BRM: χ^2 ^= 0.181, P = 0.67; FGB vs. BRM: χ^2 ^= 0.375, P = 0.54). These results indicate that none of the nuclear introns we sequenced are under selection, or that the selection regime does not differ among them. Selection on introns has recently been highlighted for birds in one locus often used in phylogenetic and phylogeographic studies [21,22], but even in such rare cases, selective appears to only act on a very few sites (e.g. 2.2% in mammals [23]).

#### FGB

We obtained the complete FGB intron-5 sequence for 98 individuals (559 bp, 25 SNPs). The allelic phase of six individuals (*debilis*: DM29193; *rabai*: ZMUC117618, ZMUC119477, ZMUC121068, ZMUC132719, ZMUC132720) could not be resolved at the 0.75 PP threshold, even when we incorporated partial sequences from museum specimens. These six individuals were excluded from further analyses; this resulted in the loss of eight SNPs, all only present in one out of 196 possible copies).

In the final data set, sixteen alleles were found in the lowland group and three in the montane group. Sharing of haplotypes between the lowland and montane population was very limited, involving only one individual: *rabai *ZMUC134311 from Mt. Kanga, with one allele assigned to *albigula *(Additional File 2)*- *morphologically this is a typical *rabai *specimen. One further bird from Mt. Kanga (ZMUC132719) resembles *albigula *in morphology but shared SNPs with both *albigula *and *rabai *(note that one autapomoprhic SNP could not be phased and this individual was thus excluded). The alleles within the lowland group (Mozambique/Zimbabwe *debilis *and Tanzania *rabai*) were inter-mixed. The results of the AMOVA indicated significant structuring of the genetic variability when partitioning by subspecies (df = 183, ϕ_st _= 0.79, P < 0.001), with most of the molecular variability being found among the three subspecies (among: 78.9%, within: 21.1%).

#### GAPDH

We obtained the complete GAPDH intron-11 sequence for 113 individuals (328 bp, 21 SNPs). One individual (ZMUC137398) had a three base pair deletion and another (ZMUC137531) had a single base pair insertion. We considered the three base pair deletion to be a single mutational event. Eight individuals (*albigula*: ZMUC132956, ZMUC132939, ZMUC132860; *rabai*: ZMUC117617, ZMUC119477, ZMUC120028, ZMUC121087, ZMUC121069) could not be phased at the threshold PP of 0.75 and were excluded from further analyses (four SNPs were only found in one out of the 226 copies, one SNP was present in two copies but only shared between two excluded individuals). We also excluded the individual which had the autapomorphic deletion of three base pairs due to difficulty in determining the allelic phase. Most of the alleles were shared among the different subspecies (Additional File 2). Yet, the AMOVA indicated significant structuring of genetic variability when partitioned by subspecies (df = 207, ϕ_st _= 0.32, P < 0.001), with most of the molecular variability being found within the three subspecies (among: 32.2%, within: 67.8%).

### Z-linked locus

We obtained complete BRM intron-15 sequences from 104 individuals (66 males, 37 females, 1 unknown; 364 bp, 14 SNPs). Twelve SNPs remained in the data set (106 individuals, 68 males, 37 females and 1 unknown, which we considered a female) after excluding the single individual (*rabai*: ZMUC132703) that could not be satisfactorily phased. No alleles were shared between *albigula *and *debilis*/*rabai *(Additional File 2). AMOVA indicated significant structuring of genetic variability when the dataset was partitioned by subspecies (df = 167, ϕ_st _= 0.699, P < 0.001), with most of the molecular variability being found among the three groups (among: 69.9%, within: 30.1%).

### STRUCTURE analysis of the nuclear data set

**Figure 3:**
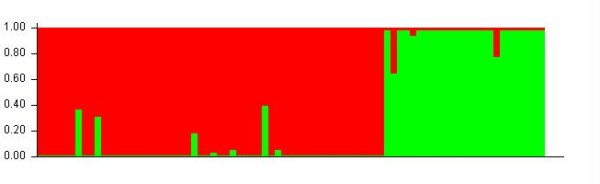
**Assignment of individuals to genetic cluster using the STRUCTURE algorithm for K = 2 (mean LogLikelihood across three runs, -ln = 595.9)**. The red color corresponds to the individuals sampled in the Nguru and Usambara Mts (*albigula*). Green corresponds to individuals sampled in the Tanzanian lowland (*rabai*) and Mozambique/Zimbabwe (*debilis*). Evidence for admixture involves five individuals sampled in the Nguru Mts, Usambara Mts and on Mt Kanga.

STRUCTURE analyses performed on the nuclear data set, revealed two primary genotype clusters (-ln P(D) = 595.9, K = 2), corresponding to the lowland (*debilis *and *rabai*) and montane (*albigula*) mtDNA clades (Figure 3). Five intermediate genotypes, with the lowest probability assignment to one or other clade varying from 0.19 to 0.40, were sampled from the East Usambara Mts (ZMUC1200079 and 120135), Nguru Mts (ZMUC132600 and 137531), and on Mt. Kanga (ZMUC134311). All the other individuals were assigned to either the montane or lowland clades with a posterior probability greater than 0.90. At K = 3, the lowland group is divided in two populations with all individuals being of mixed ancestry (assignment probability to population 2 and 3 between 0.40 and 0.60, Additional File 3). At K = 4, the pattern is very similar to the one observed at K = 2 and K = 3, but the montane group is now considered to be an admixture of two populations, which roughly correspond to the Nguru and Usambara populations.

### Multi-locus network and species tree analyses

**Figure 4:**
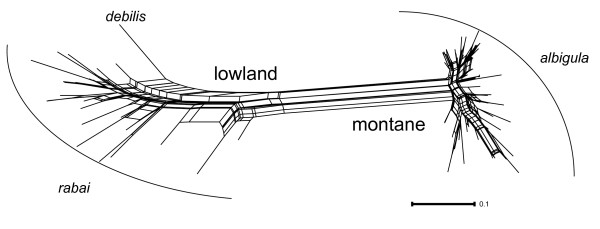
**Multi-locus network obtained using standardized genetic distances of the three nuclear loci**. Only individuals that could be sequenced and phased with a posterior probability greater than 0.75 for all three loci (n = 80) are included.

The multi-locus network derived from the combined analyses of the mitochondrial and nuclear loci recovered: 1) a clear separation of the montane (*albigula*) and lowland (*debilis *and *rabai*) clades, and 2) a greater extent of genetic diversity within the lowland clade relative to the montane clade (Figure 4).

The topology we obtained from the species tree analyses based on the coalescent approach (*BEAST) and information from the mitochondrial and nuclear loci recovered a pattern where the lowland subspecies (*debilis *and *rabai*) are sister to the montane subspecies (*albigula*), although support for the monophyly of the lowland group is limited (PP = 0.56).

The analyses performed using the species delimitation rjMCMC algorithm implemented in BPP V2.0 and a (*albigula*, (*debilis*, *rabai*)) guide tree indicates that all models visited support at least two lineages: *albigula *and *debilis*/*rabai *(PP = 1.0); this result was not dependent on the assumption of a large or small effective population size or a shallow or deep divergence as all prior combinations resulted in the same posterior probability. A speciation probability greater than 0.95 was recovered for the node leading to *debilis *and *rabai *in three of the four prior combinations; the prior combination of small effective population size and deep divergence resulted in a speciation probability of 0.76 for *debilis *and *rabai*.

### Genetic analyses: divergence time estimates

Divergence date estimates using the mitochondrial neutral mutation rate of the four-fold degenerated sites were quite variable (Table 2): from a 0.15 myrs (95% HPD: 0.02-0.5 myrs) estimate for the TMRCA of *debilis *to 3.1 myrs (95% HPD: 1.2-7.7 myrs) for the divergence between the lowland (*debilis*/*rabai*) and montane clades (*albigula*). The divergence between the lowland lineages in Tanzania (*rabai*) and Mozambique/Zimbabwe (*debilis*) is estimated to have occurred about 1.1 myrs ago (95% HPD: 0.3-2.3 myrs). The estimates obtained using a strict molecular clock hypothesis and the 6.1%/myr^-1 ^rate were very similar to the dates obtained using the neutral four-fold mutation rate and the 95% HPD were largely overlapping. In contrast, the divergence times obtained using the traditional 2.1%/myr^-1 ^clock were much older with only slight overlap with the 95% HPD obtained with the other rates (Table 2).

**Table 2:** Median estimate of the TMRCA (in million years before present) for the mtDNA data set.

Lineage	TMRCA four-fold	TMRCA 2.1%	TMRCA 6.1%
*debilis sensus lato*	3.1 (1.2-7.7)	7.7 (6.0-9.7)	2.6 (2.0-3.2)

*rabai*	0.25 (0.08-0.7)	0.8 (0.6-1.1)	0.3 (0.2-0.4)

*debilis*	0.15 (0.02-0.5)	0.6 (0.3-0.9)	0.2 (0.1-0.3)

*rabai*/*debilis*	1.1 (0.3-2.3)	2.8 (2.1-3.7)	0.9 (0.1-1.2)

*albigula*	0.4 (0.1-0.9)	0.6 (0.4-0.9)	0.2 (0.1-0.3)

Values in brackets are the 95% HPD. TMRCA were estimated using an uncorrelated lognormal molecular clock model, coalescent prior with constant population size and a mean rate of evolution for the four-fold degenerated sites of 0.073 s/s/myr (95% CI: 0.025-0.123 s/s/myr); 192 sites were included in the analyses.

### Genetic analyses: fluctuation in population size

We found evidence of population expansion in the mitochondrial data set for the Tanzanian lowland *rabai *(Fu's *Fs *= -3.976, P = 0.01, Table 1), although the R_2 _test was not significant (R_2 _= 0.0742, P = 0.10). Likewise, several significant Fu's *Fs *or R_2 _values were found for the nuclear data (e.g. GAPDH for *rabai*; *Fs *= -8.485, p < 0.01, R_2 _= 0.0596, p = NS, FGB for *albigula Fs = -*3.527, p < 0.05/R_2 _= 0.0466, p < 0.05). We observed several cases of discrepancies between the two tests, which may be due to differences in power [24]. Given our sampling of haplotypes (sample size greater than 40 in most cases), it is likely that Fu's *Fs *will have more power to detect population growth than R_2 _[24]. Finally given the difference in the test's significance across data sets and the number of segregating sites per locus (Table 1), it is very likely that the demographic expansions, when present, were moderate and not sudden [24]. The Bayesian Skyline Plot of the mitochondrial data set for the subspecies *rabai *also supports a population expansion, a three-fold increase in effective population, but the increase was continuous and not sudden (Additional File 4). The Bayesian Skyline Plot for the subspecies *albigula *did not show any evidence of population expansion (Additional File 4).

### Genetic analyses: Coalescent analyses under the Isolation with Migration model

The SBP and GARD algorithms did not detect any evidence of recombination in the three nuclear loci. Hence we used the complete sequence for the IMa analyses. Appropriate mixing and satisfactory effective sampled sizes were achieved using 12 chains and a geometric heating scheme (g1 = 0.15 and g2 = 0.70) for all parameters except *T *(time of population divergence; Table 3), where a non-zero probability tail was observed. Hence, for *T*, we considered the highest point of the posterior distribution to be the most reliable estimate.

**Table 3:** Estimates of the Isolation with Migration model using IMa.

	*RABAI*-*ALBIGULA*	*RABAI*-*ALBIGULA *KANGA EXCLUDED	*DEBILIS*-*RABAI*	*ALBIGULA*_NGURU_-*ALBIGULA*_USAMBARA_
Θ_1_	7.019 (4.870-9.659)	6.531 (4.452-9.194)	3.266 (1.594-4.9398)	1.0913 (0.465-1.705)

Θ_2_	2.426 (1.480-3.632)	2.402 (1.502-3.556)	8.355 (5.881-10.742)	1.409 (0.584-2.194)

Θ_A_	21.321 (1.587- 52.328)	25.909 (1.344-51.185)	21.068 (0.0261-44.8762)	8.6927 (0.009-16.221)

M_12_	0.109 (0.025-0.245)	0.0535 (0.005-0.145)	0.2735 (0.005-0.605)	2.8637 (0.355-5.305)

M_21_	0.123 (0.015-0.345)	0.093 (0.005-0.195)	0.186 (0.005-0.365)	1.113 (0.005-2.465)

*T*	2.4	2.2	0.6	0.3

Values represent the mean of the distribution and the 90% HPD confidence interval.

The posterior distribution of the parameter *T *possesses a non-zero probability distribution when *T *increases; the value represents the highest posterior estimate. For this reason we do not discuss the *T *value from the Isolation with Migration analyses in the text. The estimates for the *T *parameters are given in million years before present.

For the comparison between the montane populations of *albigula *from the Usambara and Nguru Mts, some models that assume no gene flow could not be rejected (2LLR = 6.7102, df = 3, ns).

For the comparison between the Tanzanian lowland and montane clades (*rabai *versus *albigula*), all models that assume no gene flow and asymmetrical gene flow, and all models that assume equal effective population sizes in the two extant lineages were strongly rejected (all P < 0.001). To determine if gene flow between *rabai *and *albigula *was primarily occurring on Mt. Kanga as suggested by the STRUCTURE assignments and morphological data, we conducted a separate IMa analysis with the individuals from Mt. Kanga excluded. With birds from Mt. Kanga excluded we could no longer reject zero gene flow taking place between *rabai *and *albigula *(with birds from Kanga 2LLR = 32.6935, df = 2, P < 0.001; without birds from Kanga 2LLR = 4.0783, df = 2, ns). This result is consistent with secondary contact and apparent introgression occurring at mid-altitude on Mt. Kanga.

For the comparison between the lowland populations from Mozambique/Zimbabwe (*debilis*) and Tanzania (*rabai*), models that assume equal population sizes (both present and past) were all rejected (least significant p-value, 2LLR = 4.5165, df = 1, P = 0.03). Models that assume no gene flow were all rejected, although one was only marginally rejected (2LLR = 8.8385, df = 3, p = 0.03). Hence, we are unable to decisively exclude the possibility that zero gene flow is occurring between Mozambique/Zimbabwe and the Tanzanian lowland populations.

The coalescent analyses between the Tanzanian montane clade (*albigula*) and the Mozambique/Zimbabwe lowland clade (*debilis*) was not performed because one of the IMa assumptions, that is, no gene flow is occurring with an unsampled lineage (geographically intermediate *rabai *in our case), was not satisfied.

The highest posterior estimate for the divergence times between populations pairs were very similar or identical to the TMRCA we obtained using the mitochondrial data set and the neutral four-fold and 6.1%/Myr^-1 ^rate (Tables 2 and 3).

### Bioclimatic data

**Figure 5:**
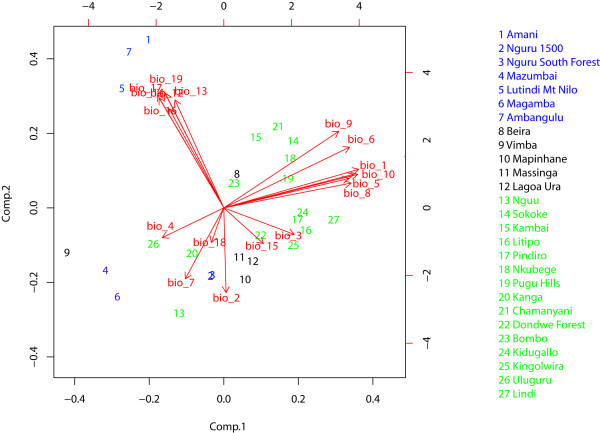
**Biplot of the first two components of the bioclimatic variables extracted from our sampling locality co-ordinates**. Note the rather disparate environmental conditions for the *albigula *sampling points. All sites where gene flow was recorded (e.g. Mt. Kanga) are characterized by reduced seasonality (variable 4).

The Principal Component Analysis performed on the bioclimatic data revealed that the lowland (*debilis*/*rabai*) and montane (*albigula*) localities could primarily be separated by the level of seasonality. The first two axes explain 92.7% of the variability (PCA Axis 1: 64.3%, PCA Axis 2: 28.4%). Sites from the Usambara Mts appear to be very heterogeneous in their distribution (Figure 5). All sites where gene flow was detected between *albigula *and *rabai *are in geographical proximity to each other (on the altitudinal gradient) and appear to have reduced seasonality, suggesting that this climatic variable may provide a key environmental context under which gene flow among the two lineages may take place.

## Discussion

### Secondary contact versus disruptive selection on an altitudinal gradient?

Birds from the montane forests of the Nguru and Usambara Mts (*albigula*) represent a distinct clade in the molecular analysis and are also readily distinguishable by their grey underparts. In addition, *albigula *is generally larger and individuals tend to have a green rather than grey crown, the later being more characteristic of lowland birds (*rabai *and *debilis*), although some *rabai *individuals do have some greenish streaks on the nape and crown. Birds of the *rabai *clade are found in foothill forests at a few hundred meters elevation in the East Usambara Mts, in the Tanzanian coastal lowlands, as well as on Mt. Kanga (common to 900 m), which rises steeply from the lowlands and is only 10 km to the east of the Nguru Mts, where *albigula *occurs (Figure 1). Our STRUCTURE analyses revealed that gene flow or introgression has occurred at three localities: on Mt. Kanga, in the foothills forests of the East Usambara Mts (Amani and Mazumbai) and in the Nguru Mts. However, Mt. Kanga is at present the only locality where we have sampled an individual with a genotype/phenotype mismatch and excluding individuals from this locality from the Isolation with Migration analyses had strong impact on the conclusion concerning gene flow between *albigula *and *rabai*.

In the Nguu Mts to the north of the Nguru Mts but partly connected by several small hills, *rabai *is found up to 1000 m in Kilindi Forest and to 1550 m in Derema Forest. This results in the montane Nguru population of *albigula *being surrounded to both the north and east by the predominantly lowland *rabai*. Thus, it appears that *rabai *is able to ascend into the submontane zone, except where this habitat is already occupied by *albigula*, which suggests that two mutually exclusive and competing taxa may be involved. It is noteworthy that birds representing the two clades can be found in close proximity to each other. We found some evidence of population expansion for *rabai *(ND2; Fu's *Fs *= -3.976, P = 0.01, GAPDH: Fu's *Fs *= -8.485, P = 0.01), suggesting that the current contact between the two taxa (Mt. Kanga, foothill forest in the East Usambara Mts) may be secondary, centered in areas of transitional habitat with low seasonality between montane and lowland forest and not the result of disruptive selection on an altitudinal gradient. The secondary contact zone hypothesis between once allopatric populations has also been favored by several other studies that have addressed this topic [e.g. [16,25]].

Our coalescent analyses under the Isolation with Migration model did allow us to reject the hypothesis of zero gene flow between the lowland (*rabai*) and highland (*albigula*) populations. Yet, when individuals from Mt. Kanga were excluded from the analyses, the hypothesis of zero gene flow could no longer be rejected. This pattern suggests that limited gene flow between *albigula *and *rabai *takes place on Mt. Kanga. Given that the lowland population likely experienced a demographic expansion, it is likely that Mt. Kanga represents a zone of secondary contact and not the initial area where the two species diverged. Consequently, recurrent gene flow appears to be restricted in space to areas of intermediate habitat. The Isolation with Migration model implemented in IMa has several assumptions that may be violated in empirical data sets, including: no intralocus recombination, no gene flow with an unsampled species/population, no linkage among loci, no selection and no population structure in the ancestral population. Here, we can reasonably reject recombination, selection and linkage among the sampled loci as potential biases. Gene flow with an unsampled species is unlikely, as the Tiny Greenbul has no close extant relatives [26]. Ancestral population structure is thought to have little effect on parameters estimates [27]. It has been shown through simulation study that using an over-simplified substitution model increases the variance in some parameter estimates [27]. The most complex substitution model implemented in IMa is HKY, which is very close to the best-fit model selected for our data set (HKY+Γ). Thus, we consider our estimates obtained with the Isolation with Migration model to not be strongly affected by currently described biases.

Given that the two lineages are well differentiated genetically and morphologically, we do not regard limited gene flow at 1-3 localities of intermediate habitat as a sufficient reason to reject species rank for the montane clade. Indeed, hybridization and introgression between 'good species' is common, especially for neutral loci and does not preclude them from being genetically distinct. (e.g. [28,29]). Hence, based on the molecular, morphological and altitudinal distribution patterns, we suggest that the taxa *albigula *and *debilis *(including *rabai*) be considered distinct species.

### Genetic structure among the highlands

The Nguru and Usambara Mts are separated by a substantial 100-160 km lowland gap of dry savannah. As these two sky islands hold the only two populations of *albigula*, it is important to know if gene flow is occurring between the two populations. Our coalescent-based analyses could not exclude the hypothesis of zero gene flow between the two populations. Our result is consistent with phylogeographic studies of other Eastern Arc Mountain birds where the Usambara-Nguru gap has consistently been recovered as the major phylogeographic break among clades [e.g. [4,8,30,31]].

In contrast to the pattern of strong genetic structuring in birds, and several other vertebrates [32,33], weak genetic differentiation (0.2%) was found between two populations of a frog, *Arthroleptis xenodactylus*, from the Usambara and Nguru Mts [34]. These results are not easy to compare as these organisms have different dispersal capacities, yet it is surprising that several montane bird lineages appear to be more geographically structured than the single frog study published to date. However, one striking pattern among all of the above studies remains that, vertebrate populations from the northern (Pare, Usambara Mts) and central mountains (Nguru, Ukaguru, Rubeho Mts) are genetically differentiated from each other across the Usambara-Nguru lowland gap. Yet, only a few of the estimated divergence times (TMRCA for *albigula *0.4 mya, 95% HPD: 0.1-0.9 mya) are compatible with each other, suggesting several periods where gene flow was interrupted among species from the same community. Hence, whereas the Pleistocene climatic oscillations likely constrained the distribution of mountain birds in the Eastern Arc due to aridification, the response of individual species to these climatic fluctuations appears to have been varied, suggesting that a model assuming a single 'common' vicariant event is unlikely [4,34].

### Genetic structure in the Tanzanian lowland (rabai spp)

Birds from Lindi District, located on the coast in SE Tanzania are more closely related to the northern Tanzanian-Kenyan lowland populations, than to the central Mozambique and Zimbabwe populations, as traditionally thought. The coastal zone of northern Mozambique is fairly dry, and there may be a significant gap in the distribution of this species. Recent surveys (2009) in NE Mozambique did not detect the species (J. Fuchs and J-M Pons, unpubl.)

Our analyses revealed substantial genetic structure and genetic diversity in the lowland clade, as inferred from the mean genetic distance among haplotypes and nucleotide diversity. Part of this genetic diversity may be explained by isolation-by-distance. Yet, we also observed substantial mitochondrial differentiation within some localities (e.g. on Mt. Kanga, Pugu and Nkubege Forests). This would either imply that the distribution of the lowland forest population has remained stable and large for a considerable period of time, or that these localities represent areas of secondary contact after population expansion from different lowland refugia. There is evidence in our data set for a signature of population expansion in the *rabai *clade, with individuals sampled from inland localities (Morogoro District, Kidugallo, Kingolwira, Nguu) primarily having haplotypes shared or derived from coastal populations. This pattern would be consistent with constant and large population size through time (and thus high genetic diversity) in coastal forests and a signature of population expansion towards the interior. The validation of this hypothesis would require further sampling from within the distributional range of *rabai*, which is challenging due to many coastal forests having been transformed by humans.

### Patterns of genetic differentiation in Mozambique/Zimbabwe (debilis ssp)

Our analyses indicate that populations from Mozambique/Zimbabwe are genetically differentiated from the lowland populations in Tanzania (Figure 2). Dating analyses using the mitochondrial data set indicate that the two lineages (*debils *vs. *rabai*) diverged about 1 mya, using the neutral four-fold rate and 6%/myr clocks, or 2.8 mya using the more conventional 2%/myr mitochondrial clock. The divergence time obtained using the four-fold degenerated rate and 6%/myr rate corresponds to a peak of aridification in Africa, a consequence of glaciation at higher latitudes [35,36]. This aridification peak, together with the general drying of Africa since the Miocene, may have altered the distribution of coastal lowland forest in northern Mozambique, thus promoting the divergence between the Mozambique/Zimbabwe and Tanzanian clades of Tiny Greenbul.

The pattern of disjunct populations distributed in lowland Tanzania and Zimbabwe/Mozambique has been observed for some other vertebrate species, although Zimbabwe is often not sampled for species associated with densely wooded habitats. Moodley and Bruford [37] found some differentiation of populations of Bushbuck (*Tragelaphus scriptus*) in this region, with diverged about 200 000 years ago.

Our analyses revealed the existence of two well-differentiated mitochondrial lineages within the Mozambique/Zimbabwe clade. The first clade consists of individuals collected in Mozambique (Beira/Mapinhane) and Zimbabwe (DM29199, Vimba). The second clade only includes individuals collected in Zimbabwe (Vimba). Thus, that one individual from Vimba (DM29199) has a mitochondrial haplotype characteristic of the Mozambique clade is intriguing, especially since the two mitochondrial lineages are rather divergent. Through several checks in the laboratory and because the collector visited Vimba five years later than central Mozambique (Dondo Beira), we can rule out sample mix-up or contamination. Therefore, the most plausible explanation is recent gene flow from central Mozambique to Vimba. This result also supports the fact that seasonality of the sites may play an important role in facilitating gene flow, as Vimba is the site with the lowest seasonality. We emphasise that further sampling in this area is needed to confirm this result.

## Conclusions

Our study suggests that the single case of altitudinal morphological segregation within a species in the Eastern Arc Mountains is likely the result of secondary contact of two highly differentiated lineages instead of disruptive selection across an altitudinal gradient. Our analyses support three main lineages within *Phyllastrephus debilis sensu lato *and stress the recognition of two species of 'Tiny Greenbul', *P. albigula *restricted to the Nguru and Usambara Mts, and *P. debilis *distributed in the Tanzanian lowland forest (ssp *rabai*) and in Mozambique/Zimbabwe (ssp *debilis*). Introgression of some alleles (mostly from *rabai *to *albigula*) appears to have occurred at three different localities (Mt. Kanga, East Usambara foothills and the Nguru Mts), in mid-altitude areas with a transition between foothill and montane forest, especially in areas which experience low seasonality. The extent of this introgression is limited and appears to be the result of the population expansion of the coastal *rabai *clade further inland. The pattern of genetic divergence we found for the montane clade (Nguru versus Usambara) is similar to what has been described in other vertebrates for mtDNA (reciprocally monophyletic), although our results from the nuclear genome emphasize the need for more studies from East Africa to include a diverse set of markers in phylogeographic studies. Greater genetic variability was recovered in the Tanzanian lowland clade, suggesting a greater long-term effective population size or the existence of distinct refugia in the past, which have now merged as lowland populations expanded.

## Methods

### Tissue and morphological data collection

Our morphometric data was drawn from 29 specimens deposited in the Zoological Museum, University of Copenhagen, as well as from notes on an additional 61 specimens deposited at other institutions (Field Museum, National Museum of Natural History, National Museums of Kenya, and Museum Alexander König). All morphological measurements and the scoring of plumage patterns were undertaken by J. Fjeldså: wing-length (flattened-chord, from carpal joint to tip of the longest primary feather) was measured to the nearest 0.1 mm using a wing-rule; weight was measured using a Pesola spring scale to the nearest 0.1 g; bill-length (from the bill tip to the base of the exposed bill on the skull), tail-length (from the insertion point on the pygostyle to the tip of the longest feather) and tarsus-length were measured using Vernier calipers to the nearest 0.1 mm. Plumage colors were matched to known color standards [38] to objectively define colors across specimens.

We collected tissue from 124 individuals (60 *albigula*, 26 *debilis *and 38 *rabai*, altogether 59 vouchers) that span the entire distributional range of the species (Additional File 5, Figure 1): 96 of the samples were fresh tissues and 28, including 24 *debilis *samples, were obtained from museum specimens (toe-pads).

### Morphological analyses

Morphological measurements were log-transformed (log x+1) to reduce variance between characters. Differences in univariate measures between taxa were tested using paired t-tests. Principal component analysis (PCA) was used to summarize morphological variation. PCA was performed on individuals and only principal components (PCs) with eigenvalues greater than one were extracted. The factor matrix was rotated using the varimax method to optimize variable loadings. The resulting rotated factor matrix was used to determine which of the original variables were most highly correlated with the PCs.

### Laboratory procedures

DNA was extracted from tissue or blood using the Qiagen extraction kit (Valencia, CA) following the manufacturer's protocol. We extracted the DNA from toe-pads in a room dedicated to ancient DNA labwork. We used the same extraction protocols as for the fresh samples, but added 20 μl of dithiothreitol (DTT, 0.1 M) to facilitate the digestion of these tissues. We amplified and sequenced four loci, one mitochondrial (ND2), two autosomal (Beta-fibrinogen intron-5, FGB; GAPDH intron-11, GAPDH) and one Z-linked (BRM intron-15, BRM). PCR-amplification was performed using standard protocols, only the annealing temperature varied (54-60°C). Locations on the chicken genome and primer sequences used are detailed in the Additional File 6. All sequences have been deposited in GenBank (Accession Numbers HQ716721-HQ717145)

### Molecular sexing

We sexed individuals with the primer pair P2/P8 using the protocol described in Griffiths et al. [39]; sex was deduced based on the number of bands (two for females and one for male). For the museum specimens, sex was inferred from the specimen labels. We could not obtain any PCR-product using the P2/P8 primer pair for 14 individuals that were homozygous at the Z-linked locus we sequenced (BRM). For these 14 individuals, we aimed to PCR-amplify and sequence three further Z-linked loci (CHDZ, ACO1, SPIN1, [40]). Our success with PCR-amplification was variable; we considered an individual to be a female if no heterozygous position was detected in at least two loci (minimum length of Z-linked data: 834 bp, maximum length of Z-linked data: 2849 bp).

### Phylogenetic reconstruction

Molecular phylogenies were estimated using maximum likelihood and Bayesian inference, as implemented in PHYML 3.0 [41] and MRBAYES 3.1.2 [42], respectively. The most appropriate models of nucleotide substitution were determined with DT_MODSEL [43]. Two analyses of four Metropolis-coupled MCMC chains (one cold and three heated) were run for ten million iterations with trees sampled every 100 iterations. The number of iterations discarded before the posterior probabilities were calculated varied among analyses. We checked that the potential scale reduction factor (PSRF) approached 1.0 for all parameters and that the average standard deviation of split frequencies converged towards zero. We also used TRACER v1.5 [44] to ascertain whether our sampling of the posterior distribution had reached a sufficient effective sample size (ESS) for meaningful parameter estimation. We made use of sequences of *P. hypochloris *(DQ402215) and *P. xavieri *(DQ402219) as outgroups.

### Testing for selection

To test whether selection was acting on the mitochondrial protein-coding gene (ND2), we used the McDonald-Kreitman test [45]. The stop codon was excluded from the analyses, leaving a total of 1038 bp for 110 Tiny Greenbuls. We used sequences of *P*. *hypochloris *(DQ402215) and *P. xavieri *(DQ402219) as outgroups. Significance was assessed using Fischer's exact test at a threshold of 0.05.

We tested for selection acting on the nuclear loci by using the HKA test [46], as implemented in DNASP 5.0 [47]. We used sequences from one Yellow-Streaked Greenbul *Phyllastrephus flavostriatus *as an outgroup (S. Lokugalappatti unpubl. data).

### Determining the phase of alleles

We used PHASE V2.1.1 [48] to infer the alleles for each nuclear locus. Three runs, using different seed values, were performed and results were compared across runs. We used the recombination model and ran the iterations of the final run 10 times longer than for the other runs. We used a threshold of 0.75 to consider a SNP to be satisfactorily phased [49] and individuals that did not satisfy this threshold were removed from further analysis.

### Population genetic analyses

Haplotype diversity (Hd), nucleotide diversity (π) and Watterson's theta (θ) were estimated with DNASP 5.0. We used TCS 1.21 [50] to reconstruct a 95% statistical parsimony network. Overall genetic structure of populations was investigated with an analysis of molecular variance (AMOVA, 1000 permutations) using pairwise distances (ϕ_st_, [51]), as implemented in ARLEQUIN v3.1 [52]. In order to test for any correlation between geographic (shortest straight line) and genetic distances (ND2 uncorrected-*p*) we performed a Mantel test in GENALEX 6 [53].

We used Fu's *Fs *test (1000 replicates) and Ramos-Onsins and Rozas R_2 _statistic [24], as implemented in DNASP 5.0, to detect signatures of demographic change. The significance of the R_2 _statistic was assessed using 1000 coalescent simulations. We also used Bayesian skyline plots [54] on the mitochondrial data set for the *albigula *and *rabai *lineages to estimate more complex scenarios of population dynamics. This method is independent of *a priori *defined demographic models and tree reconstructions, and is thus suitable for taxa with complicated population history. Analyses were run in BEAST 1.5.4. [55] using the HKY model and a strict molecular clock. The MCMC simulations were run for 20^6 ^iterations, with genealogies and model parameters being sampled every 1000 iterations. The Bayesian skyline plots (BSPs) were visualized in TRACER V.1.5 [44].

### mtDNA divergence times

Inferring divergence times within species is a challenging task as no internal fossil calibration points can be used for most species. The existence of a time dependency of substitution rates appears to now be well accepted [e.g. [56]] although its magnitude continues to be debated [57,58]. Subramanian et al. [59] suggested that the time dependency phenomenon could primarily be attributed to non-synonymous substitutions. They estimated the mean rate of evolution at four-fold degenerated sites from complete mtDNA sequences of Adelie Penguins (*Pygoscelis adeliae*) to be 0.073 (95% HPD: 0.025-0.123 s/s/myr). We applied this mutation rate, and the associated uncertainty, to the four-fold degenerated sites within our mitochondrial data set (192 sites). We used BEAST 1.5.4 [55,60] with an uncorrelated lognormal molecular clock model, coalescent tree prior with constant population size and a GTR+Γ model of sequence evolution: the same model of sequence evolution used by Subramanian et al. [57] to estimate the rate. We compared this new molecular rate with a molecular clock rate for ND2 (6.2%/Ma) proposed by Arbogast et al. [61] and the 'standard' avian mtDNA rate of 2.1% divergence per million years. MCMC chains were run for 5*10^6 ^steps and were sampled every 1000 steps.

### Population assignment using STRUCTURE

We used STRUCTURE V2 [62] to infer how many populations could be distinguished based on the three nuclear loci. We only included individuals (*n *= 80) for which: 1) all three nuclear loci could be satisfactorily phased, and 2) sequences of the three nuclear loci were available. We compared the optimal number of populations estimated by STRUCTURE and the probability of each individual being assigned to each population across analyses. We assumed an admixture model with correlated allele frequencies and let alpha vary among populations. We ran 2*10^6 ^iterations (burnin: 2*10^5 ^iterations) from K = 1 to K = 5. The number of clusters (populations) was estimated using ΔK [63].

### Multi-locus network and species tree approaches

We used POFAD V1.03 [64] and SPLITSTREE V4.0 [65] to build a multi-locus network based on the mitochondrial and nuclear data sets. We used the same set of individuals that were used in the STRUCTURE analyses. We used uncorrected-*p *distances as input for POFAD and made use of the standardized matrix for network reconstruction.

We used the species tree approach (*BEAST, [66]) implemented in BEAST 1.5.4 [55,60], to estimate the relationship among the three subspecies (*albigula*, *debilis *and *rabai*). Species tree approaches implement the coalescent to estimate a species tree based on the individual gene trees; this approach has been shown to outperform the traditional concatenation approaches in that incomplete lineage sorting is explicitly taken into account. We defined each subspecies as a 'species'. We used all individuals for which full phase information was available and assumed a strict molecular clock model for all loci and made used of the best-fit model for each partition, as determined with DT_MODSEL[43]. Thus, each locus had its own model and clock rate specified. We ran the MCMC chains for 100 million iterations.

We made use of the software BAYESIAN PHYLOGENY AND PHYLOGEOGRAPHY V2.0 (BPP v2.0, [67,68]) to estimate the speciation probability for the case where all three subspecies are considered species. This decision was based on the results from the STRUCTURE analyses, multi-locus network and species tree analyses. The method implemented in BPP v2.0 accommodates the species phylogeny as well as lineage sorting due to ancestral polymorphism. A speciation probability of 1 on a node indicates that every species delimitation model visited by the reverse-jump MCMC algorithm supports the marginal posterior probability inference that two lineages descend from a particular node as 'species'. We consider to speciation probability values greater than 0.95 as strong-support for the occurrence of a speciation event. A gamma prior was used on the population size parameters (θs) and the age of the root in the species tree (τ_0_), whereas the other divergence time parameters were assigned a Dirichlet prior [[68]: equation 2]. We ran the rjMCMC analyses for 500 000 generations with a burn-in period of 10 000 and different starting seeds. We ran each analysis at least twice. We evaluated the influence of the priors on the posterior probabilities by changing the priors for θ and τ0, assuming either small or large ancestral population size with G set to (2, 2000) and (1, 10), respectively, and shallow or deep divergence with G set to (2, 2000) and (1, 10), respectively.

### Coalescent analyses using the Isolation with Migration model

We used the Markov chain Monte Carlo method implemented in the program IMa [69] to fit the data to a model that included both isolation and migration. IMa estimates six parameters scaled to the neutral mutation rate (μ): θ_pop1 _(4Ne_pop1_μ), θ_pop_2 (4Ne_pop2_μ), θ_popA _(4Ne_popA_μ), t (T/μ, where T is the time since population divergence in years before present), m1 (2 M/θ_pop1_, where M is the effective number of migrants moving into population 1) and m2 (2 M/θ_pop2_, where M is the effective number of migrants moving into population 2). We defined inheritance scales to reflect the difference in modes of inheritance among the loci used: 0.25 for the mtDNA locus, 0.75 for the Z-linked locus and 1.0 for the two autosomal loci. We used an HKY model of nucleotide substitution for the mtDNA and an infinite-sites model for each of the three nuclear loci. Parameters and genealogies were sampled every 100 steps for the 5 and 10 million step runs. To assess convergence we monitored the extent of autocorrelation, parameter trend lines and the effective sample size (ESS) throughout the run and we also compared the results between three independent runs.

We used a generation time of 1.7 years, which reflects the average for several passerine species [70], and a mutation rate of 1.05*10^-8 ^substitutions/site/year (s/s/y) for mtDNA (6.1% per million years, [61]), and thus a per locus rate of 3.17*10^-5 ^substitutions per year. For the Z-linked locus we assumed a mutation rate of 3.6*10^-9 ^s/s/y and for autosomal loci we selected a rate of 3.61*10^-9 ^s/s/y [71]. This translated into per locus rates of: FGB 2.01*10^-6 ^s/l/y, GAPDH 1.17*10^-6 ^s/l/y, and BRM 1.31*10^-6 ^s/l/y. The geometric mean of the combined mtDNA and ncDNA was 3.14*10^-6 ^s/l/y. We tested for intralocus recombination using the GARD and SBP algorithm, as implemented in HYPHY [72,73].

We performed four pairwise comparisons: 1) *albigula *versus *rabai*, 2) *albigula *versus *rabai *with individuals collected on Mt Kanga excluded (see results), 3) *rabai *versus *debilis*, and 4) *albigula*_Nguru _versus *albigula*_Usambara_.

### Bioclimatic data analyses

Current bioclimatic data (Bioclim variables 1-18, 30 arc-seconds resolution = c. 1 × 1 km) were collected from WORLDCLIM http://www.worldclim.org/current and compiled using DIVA-GIS v7.2 [74]. The climatic envelope for each of the sampling points was then extracted. We performed a Principal Component Analysis on the 18 bioclimatic parameters extracted from each sampling point using a correlation matrix approach in R 2.10.1 [75].

## Authors' contributions

J Fuchs performed the laboratory work, carried out the genetic and bioclimatic analyses and drafted the manuscript. J Fjeldså participated in the design of the study, took the biometric and plumage data and drafted the manuscript. RB participated in the design of the study, performed the biometric and some genetic analyses and drafted the manuscript. All authors read and approved the final manuscript.

## Supplementary Material

Additional file 1 **Scatterplot of the principal component scores derived from five morphological measures**. Scatterplot of the principal component scores derived from five morphological measures for *P. debilis*. Code Key: red (1) lowland *rabai*, green (2) montane *albigula *from the Usambara Mts, and blue (3) montane *albigula *from the Nguru Mts). Click here for file

Additional file 2 **Haplotype network obtained from each nuclear loci**. Haplotype network obtained from each nuclear loci using TCS. Color codes are: *P. d. rabai *(green), *P. d. debilis *(red) and *P. d. albigula *(blue). Circle size is proportional to haplotype frequency. Note that the scale is different for each locus. Click here for file

Additional file 3 **Assignment of individuals to genetic clusters using the STRUCTURE algorithm**. Assignment of individuals to genetic clusters using the STRUCTURE algorithm for K = 3 (mean LogLikelihood across three runs, -ln = 618.7), K = 4 (mean LogLikelihood across three runs, -ln = 631.7), K = 5 (mean LogLikelihood across three runs, -ln = 602.4). Click here for file

Additional file 4 **Bayesian Skyline Plot of the mitochondrial data sets for the subspecies *rabai *and *albigula***. Bayesian Skyline Plot of the mitochondrial data sets for the subspecies *rabai *and *albigula*. Click here for file

Additional file 5 **List of samples used and Genbank Accession Numbers**. List of samples used and Genbank Accession Numbers. Click here for file

Additional file 6 **List of loci sequenced and primers used for PCR amplification and sequencing**. List of loci sequenced and primers used for PCR amplification and sequencing. Click here for file

## References

[B1] Myers N Mittermeier RA Mittermeier CG da Fonseca GA Kent J Biodiversity hotspots for conservation priorities Nature 2000 403 853 858 10.1038/35002501 10706275

[B2] Burgess ND Butynski TM Cordeiro NJ Doggart NH Fjeldsa J Howell KM Kilahama FB Loader SP Lovett JC Mbilinyi B Menegon M Moyer DC Nashanda E Perkin A Rovero F Stanley WT Stuart SN The biological importance of the Eastern Arc mountains of Tanzania and Kenya Biological Conservation 2007 134 209 231 10.1016/j.biocon.2006.08.015

[B3] Jetz W Rahbek C Colwell RK The coincidence of rarity and richness and the potential historical signature of centers of endemism Ecology Letters 2004 7 1180 1191 10.1111/j.1461-0248.2004.00678.x

[B4] Fjeldså J Bowie RCK New perspectives on the origin and diversification of Africa's forest avifauna African Journal of Ecology 2008 46 235 247 10.1111/j.1365-2028.2008.00992.x

[B5] Marchant R Mumbi C Behera S Yamagata T The Indian Ocean dipole - the unsung driver of climatic variability in East Africa African Journal of Ecology 2007 45 4 16 10.1111/j.1365-2028.2006.00707.x

[B6] Lovett JC Elevational and latitudinal changes in tree associations and diversity in the Eastern arc Mountains of Tanzania Journal of Tropical Ecology 1996 12 629 650 10.1017/S0266467400009846

[B7] Lovett JC Tanzanian forest tree plot diversity and elevation Journal of Tropical Ecology 1999 15 689 694 10.1017/S0266467499001108

[B8] Bowie RCK Fjeldså J Kiure J Multi-locus molecular DNA variation in Winifred's Warbler *Scepomycter winifredae *suggests cryptic speciation and the existence of a threatened species in the Rubeho-Ukaguru Mountains of Tanzania Ibis 2009 151 709 719 10.1111/j.1474-919X.2009.00954.x

[B9] Voelker G Outlaw RK Bowie RCK Pliocene forest dynamics as a primary driver of African bird speciation Global Ecology and Biogeography 2010 19 111 121 10.1111/j.1466-8238.2009.00500.x

[B10] Bowie RCK Fjeldså J Hackett SJ Bates JM Crowe TM Coalescent models reveal the relative roles of ancestral polymorphism, vicariance and dispersal in shaping phylogeographical structure of an African mountain forest robin Molecular Phylogenetics and Evolution 2006 38 171 188 10.1016/j.ympev.2005.06.001 16024259

[B11] Fjeldså J Bowie RCK Kiure J The Forest Batis, *Batis mixta*, is two species: description of a new, narrowly distributed *Batis *species in the Eastern Arc biodiversity hotspot Journal of Ornithology 2006 147 578 590 10.1007/s10336-006-0082-4

[B12] Poynton JC Loader SP Clinal variation and its taxonomical consequences in the common Tanzanian forest frog, *Arthroleptis affinis* Copeia 2008 2008 17 526

[B13] Dickinson ED The Howard and Moore Complete Checklist of the Birds of the World 2003 third Princeton University Press, Princeton, New Jersey

[B14] Romdal TS Altitudinal distribution and abundance patterns of bird species in the Eastern Arc Mountains, Tanzania Scopus 2001 21 35 54

[B15] Patton JL Smith MF MtDNA phylogeny of Andean mice: A test of diversification across ecological gradients Evolution 1992 46 174 183 10.2307/2409812 28564953

[B16] García-Moreno J Fjeldså J Rheinwald G Chronology and mode of speciation in the Andean avifauna Isolated vertebrate communities in the tropics. Proceedings of the 4th International Symposium: Isolated vertebrate communities in the tropics, Bonn 2003 25 46

[B17] McCormack JE Smith TB Niche expansion leads to small-scale adaptive divergence along an elevation gradient in a medium-sized passerine bird Proceedings of the Royal Society of London, B 2008 275 2155 2164 10.1098/rspb.2008.0470 PMC2603212 18544512

[B18] Schluter D Evidence for ecological speciation and its alternative Science 2009 323 737 741 10.1126/science.1160006 19197053

[B19] Orr HA Speciation 2004 Sinauer & Associates, Sunderland, Massachussets

[B20] Keith S Urban EK Fry CH The Birds of Africa Acad Press London 1992 IV

[B21] Schroeder J Kentie R van der Velde M Hooijmeijer JCEW Both C Haddrath O Baker AJ Piersma T Linking intronic polymorphism on the CHD1-Z gene with fitness correlates in Black-tailed Godwits *Limosa l. limosa* Ibis 2010 152 368 377 10.1111/j.1474-919X.2009.01005.x

[B22] Benedict L Bowie RCK Fuchs J McManes M When non-coding is non-neutral: the role of CHD1 gene polymorphism in sexing, phylogenetics and as a correlate of fitness in birds Ibis 2010 152 223 225 10.1111/j.1474-919X.2010.01015.x

[B23] Pollard KS Hubisz MJ Rosenbloom KR Siepel A Detection of nonneutral substitution rates on mammalian phylogenies Genome Research 2010 20 110 121 10.1101/gr.097857.109 19858363 PMC2798823

[B24] Ramos-Onsins R Rozas R Statistical properties of new neutrality tests against population growth Molecular Biology and Evolution 2002 192 2092 2100 10.1093/oxfordjournals.molbev.a004034 12446801

[B25] Dingle C Lovette IJ Canaday C Smith TB Elevational zonation and the phylogenetic relationships of the *Henicorhina *wood-wrens Auk 2006 123 119 134 10.1642/0004-8038(2006)123[0119:EZATPR]2.0.CO;2

[B26] Johansson US Fjeldså J Lokugalappatti LGS Bowie RCK A nuclear DNA phylogeny and proposed taxonomic revision of African greenbuls (Aves, Passeriformes, Pycnonotidae) Zoologica Scripta 2007 36 417 427 10.1111/j.1463-6409.2007.00290.x

[B27] Strasburg JL Rieseberg LH How robust are "isolation with migration" analyses to violations of the im model? A simulation study Molecular Biology and Evolution 2010 27 297 310 10.1093/molbev/msp233 19793831 PMC2877552

[B28] Carling MD Brumfield RT Haldane's rule in an avian system: using cline theory and divergence population genetics to test for differential introgression of mitochondrial, autosomal and sex-linked loci across the *Passerina *bunting hybrid zone Evolution 2008 62 2600 2615 10.1111/j.1558-5646.2008.00477.x 18691261

[B29] Yuri T Jernigan RW Brumfield RT Bhagabati NK Braun MJ The effect of marker choice on estimated levels of introgression across an avian (Pipridae *Manacus*) hybrid zone Molecular Ecology 2009 18 4888 4903 10.1111/j.1365-294X.2009.04381.x 19863717

[B30] Bowie RCK Fjeldså J Hackett SJ Crowe TM Systematics and biogeography of Double-Collared Sunbirds from the Eastern Arc Mountains, Tanzania Auk 2004 121 660 681 10.1642/0004-8038(2004)121[0660:SABODS]2.0.CO;2

[B31] Bowie RCK Voelker G Fjeldså J Lens L Hackett SJ Crowe TM Systematics of the Olive Thrush *Turdus olivaceus *species complex with reference to the taxonomic status of the endangered Taita Thrush *T. helleri* Journal of Avian Biology 2005 36 391 404 10.1111/j.0908-8857.2005.03459.x

[B32] Gravlund P Molecular phylogeny of Tornier's cat snake (*Crotaphopeltis tornieri*), endemic to East African mountain forests: biogeography, vicariance events and problematic species boundaries Journal of Zoological Systematics and Evolutionary Research 2002 40 46 56 10.1046/j.1439-0469.2002.00175.x

[B33] Stanley WT Olson LE Phylogeny, phylogeography, and geographic variation of *Sylvisorex howelli*, an endemic shrew of the Eastern Arc Mountains Journal of Zoology 2005 266 341 354 10.1017/S0952836905007016

[B34] Blackburn DC Measey GJ Dispersal to or from an African biodiversity hotspot? Molecular Ecology 2009 18 1904 1915 10.1111/j.1365-294X.2009.04156.x 19434809

[B35] de Menocal PB Plio-Pleistocene African climate Science 1995 270 53 59 10.1126/science.270.5233.53 7569951

[B36] de Menocal PB African climate change and faunal evolution during the Pliocene-Pleistocene Earth Planetary Science Letters 2004 220 3 24 10.1016/S0012-821X(04)00003-2

[B37] Moodley Y Bruford MW Molecular biogeography: towards an integrated framework for conserving Pan-African biodiversity PLos One 2007 2 e454 10.1371/journal.pone.0000454 17520013 PMC1866246

[B38] Ridgway R Color Standards and Color Nomenclature 1912 United States National Museum, Washington, D.C

[B39] Griffiths R Double MC Orr K Dawson RJG A DNA test to sex most birds Molecular Ecology 1998 7 1071 1075 10.1046/j.1365-294x.1998.00389.x 9711866

[B40] Kimball RT Braun EL Barker FK A well-tested set of primers to amplify regions spread across the avian genome Molecular Phylogenetics and Evolution 2009 50 654 660 10.1016/j.ympev.2008.11.018 19084073

[B41] Guindon S Gascuel O A simple, fast and accurate algorithm to estimate large phylogenies by maximum likelihood Systematic Biology 2003 52 696 704 10.1080/10635150390235520 14530136

[B42] Ronquist F Huelsenbeck JP MrBayes 3: Bayesian phylogenetic inference under mixed models Bioinformatics 2003 19 1572 1574 10.1093/bioinformatics/btg180 12912839

[B43] Minin V Abdo Z Joyce P Sullivan J Performance-based selection of likelihood models for phylogeny estimation Systematic Biology 2003 52 1 10 10.1080/10635150390132632 14530134

[B44] Rambaut A Drummond AJ Tracer v1.5 2007 http://tree.bio.ed.ac.uk/software/tracer/

[B45] McDonald JH Kreitman M Adaptive protein evolution at the Adh locus in *Drosophila* Nature 1991 351 652 654 10.1038/351652a0 1904993

[B46] Hudson RR Kreitman M Aguadé M A test of neutral molecular evolution based on nucleotide data Genetics 1987 116 153 159 3110004 10.1093/genetics/116.1.153 PMC1203113

[B47] Librado P Rozas J DnaSP v5: a software for comprehensive analyis of DNA polymorphism data Bioinformatics 2009 25 1451 1452 10.1093/bioinformatics/btp187 19346325

[B48] Stephens M Smith NJ Donnelly P A new statistical method for haplotype reconstruction from population data American Journal of Human Genetics 2001 68 978 989 10.1086/319501 11254454 PMC1275651

[B49] Harrigan RJ Mazza ME Sorenson MD Computation vs. cloning: evaluation of two methods for haplotype determination Molecular Ecology Resources 2008 8 1239 1248 10.1111/j.1755-0998.2008.02241.x 21586011

[B50] Clement M Posada D Crandall KA TCS: a computer program to estimate gene genealogies Molecular Ecology 2000 9 1657 1659 10.1046/j.1365-294x.2000.01020.x 11050560

[B51] Excoffier L Smouse P Quattro J Analysis of molecular variance inferred from metric distances among DNA haplotypes: application to human mitochondrial DNA restriction data Genetics 1992 131 479 491 1644282 10.1093/genetics/131.2.479 PMC1205020

[B52] Excoffier L Laval G Schneider S Arlequin ver. 3.0: An integrated software package for population genetics data analysis Evolutionary Bioinformatics Online 2005 1 47 50 19325852 PMC2658868

[B53] Peakall R Smouse PE GENALEX 6: genetic analysis in Excel. Population genetic software for teaching and research Molecular Ecology Notes 2006 6 288 295 10.1111/j.1471-8286.2005.01155.x PMC3463245 22820204

[B54] Drummond AJ Rambaut A Shapiro B Pybus OG Bayesian coalescent inference of past population dynamics from molecular sequences Molecular Biology and Evolution 2005 22 1185 1192 10.1093/molbev/msi103 15703244

[B55] Drummond AJ Rambaut A BEAST: Bayesian evolutionary analysis by sampling trees BMC Evolutionary Biology 2007 7 214 10.1186/1471-2148-7-214 17996036 PMC2247476

[B56] Ho SHW Shapiro B Phillips MJ Cooper A Drummond AJ Evidence for time dependency of molecular rate estimates Systematic Biology 2007 56 515 522 10.1080/10635150701435401 17562475

[B57] Debruyne R Poinar HN Time dependency of molecular rates in ancient DNA data sets, a sampling artefact Systematic Biology 2009 58 348 360 10.1093/sysbio/syp028 20525589

[B58] Navascués M Emerson BC Elevated substitution rate estimates from ancient DNA: model violation and bias of Bayesian methods Molecular Ecology 2009 18 4330 4397 10.1111/j.1365-294X.2009.04318.x 19735451

[B59] Subramanian S Denver DR Millar CD Heupink T Aschrafi A Emslie SD Baroni C Lambert DM High mitogenomic evolutionary rates and time dependency Trends in Genetics 2009 25 482 486 10.1016/j.tig.2009.09.005 19836098

[B60] Drummond AJ Ho SYW Phillips MJ Rambaut A Relaxed phylogenetics and dating with confidence PLoS Biology 2006 4 e88 10.1371/journal.pbio.0040088 16683862 PMC1395354

[B61] Arbogast BS Drovetski SV Curry RL Boag PT Seutin G Grant PR Grant BR Anderson D The origin and diversification of Galapagos mockingbirds Evolution 2006 60 370 382 16610327

[B62] Falush D Stephens M Pritchard JK Inference of population structure using multi-locus genotype data: linked loci and correlated allele frequencies Genetics 2003 164 1567 1587 12930761 10.1093/genetics/164.4.1567 PMC1462648

[B63] Evanno G Regnaut S Goudet J Detecting the number of clusters of individuals using the software STRUCTURE: a simulation study Molecular Ecology 2005 14 2611 2620 10.1111/j.1365-294X.2005.02553.x 15969739

[B64] Joly S Bruneau A Incorporating allelic variation for reconstructing the evolutionary history of organisms from multiple genes: an example from *Rosa *in North America Systematic Biology 2006 55 623 636 10.1080/10635150600863109 16969938

[B65] Huson DH Bryant D Application of phylogenetic networks in evolutionary studies Molecular Biology and Evolution 2006 23 254 267 16221896 10.1093/molbev/msj030

[B66] Heled J Drummond AJ Bayesian inference of species trees from multilocus data Molecular Biology and Evolution 2010 27 570 580 10.1093/molbev/msp274 19906793 PMC2822290

[B67] Rannala B Yang Z Bayes Estimation of species divergence times and ancestral population sizes using DNA sequences from multiple loci Genetics 2003 164 1645 1656 12930768 10.1093/genetics/164.4.1645 PMC1462670

[B68] Yang Z Rannala B Bayesian species delimitation using multilocus sequence data Proceedings of the National Academy of Sciences, USA 2007 107 9264 9269 10.1073/pnas.0913022107 PMC2889046 20439743

[B69] Hey J Nielsen R Integration within the Felsenstein equation for improved Markov chain Monte Carlo methods in population genetics Proceedings of the National Academy of Sciences, USA 2007 104 2785 2790 10.1073/pnas.0611164104 PMC1815259 17301231

[B70] Sæther B-E Lande R Engen S Generation time and temporal scaling of bird population dynamics Nature 2005 436 99 102 10.1038/nature03666 16001068

[B71] Axelsson E Smith NGC Sundström H Berlin S Ellegren H Male-biased mutation rate and divergence in autosomal, Z-Linked and W-Linked introns of chicken and turkey Molecular Biology and Evolution 2006 21 1538 1547 10.1093/molbev/msh157 15140948

[B72] Kosakovsky Pond SL Frost SDW Muse SV HyPhy: hypothesis testing using phylogenies Bioinformatics 2005 21 676 679 15509596 10.1093/bioinformatics/bti079

[B73] Kosakovsky Pond SL Posada D Gravenor MB Woelk CH Frost SDW GARD: A Genetic Algorithm for Recombination Detection Bioinformatics 2006 22 3096 3098 10.1093/bioinformatics/btl474 17110367

[B74] Hijmans RJ Cameron SE Parra JL Jones PG Jarvis A Very high resolution interpolated climate surfaces for global land areas International Journal of Climatology 2005 25 1965 1978 10.1002/joc.1276

[B75] R Development Core Team R: A language and environment forstatistical computing 2008 R Foundation for Statistical Computing, Vienna, Austria http://www.R-project.org ISBN 3-900051-07-0

