# Supplementary figures and images for: Diversification across an altitudinal gradient in the Tiny Greenbul (Phyllastrephus debilis) from the Eastern Arc Mountains of Africa
Source: BMC Evol Biol. 2011 May 3;11:117. doi: 10.1186/1471-2148-11-117 (PMC3097164; doi:10.1186/1471-2148-11-117)

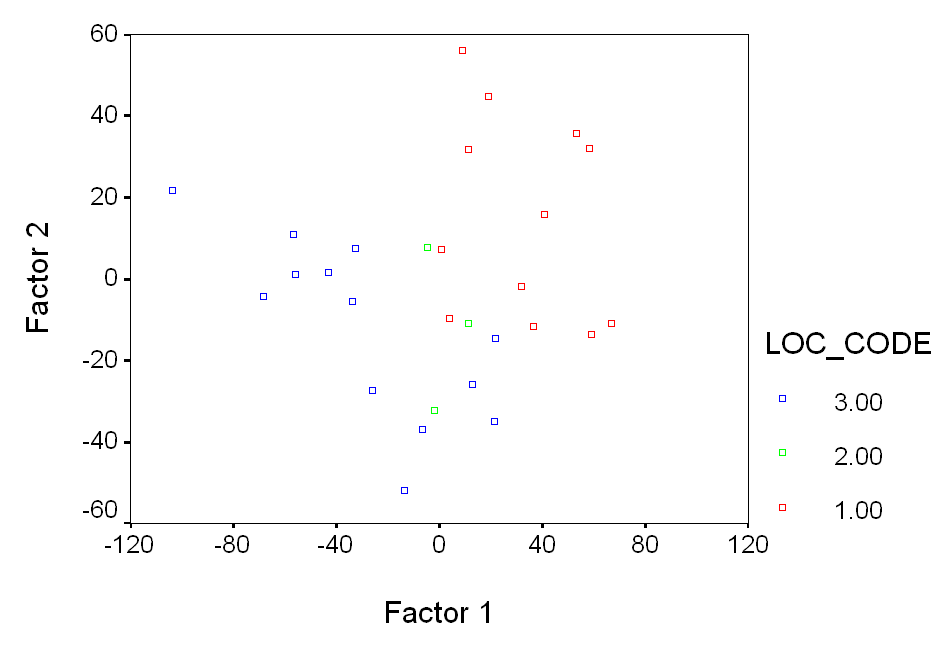

Supplement: Additional file 1 — Scatterplot of the principal component scores derived from five morphological measures. Scatterplot of the principal component scores derived from five morphological measures for P. debilis. Code Key: red (1) lowland rabai, green (2) montane albigula from the Usambara Mts, and blue (3) montane albigula from the Nguru Mts). [file 1471-2148-11-117-S1.TIFF]

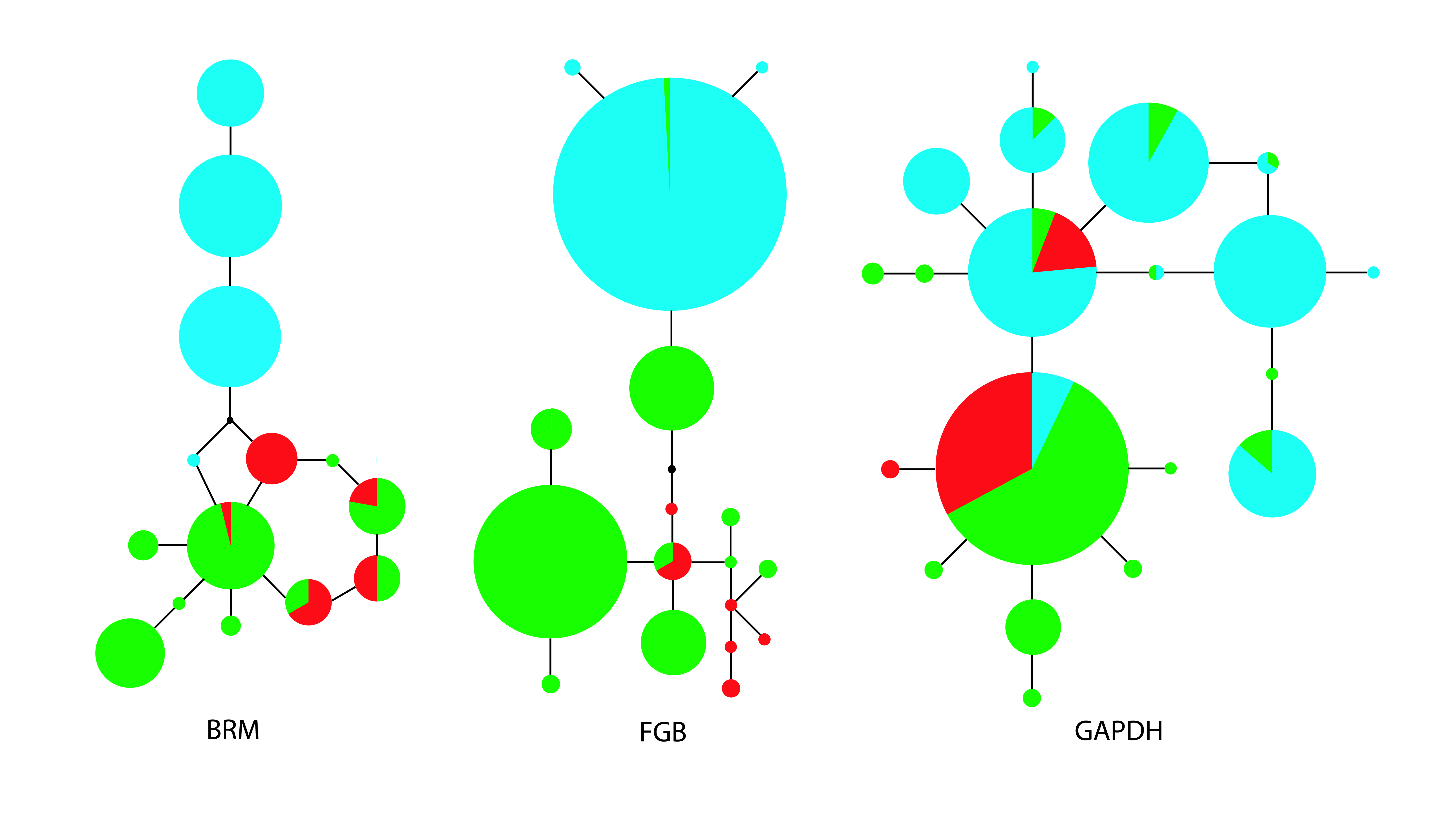

Supplement: Additional file 2 — Haplotype network obtained from each nuclear loci. Haplotype network obtained from each nuclear loci using TCS. Color codes are: P. d. rabai (green), P. d. debilis (red) and P. d. albigula (blue). Circle size is proportional to haplotype frequency. Note that the scale is different for each locus. [file 1471-2148-11-117-S2.JPEG]

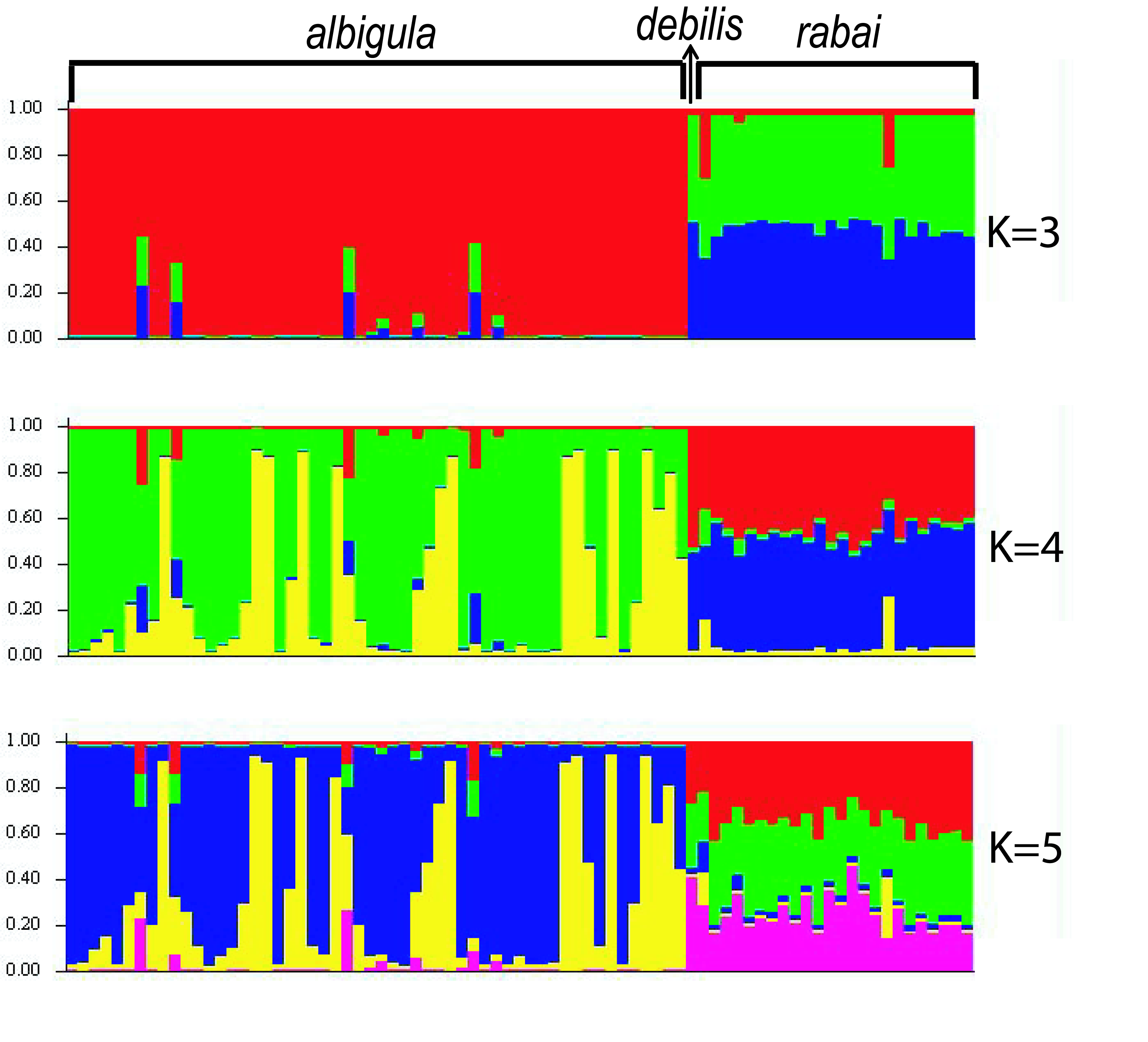

Supplement: Additional file 3 — Assignment of individuals to genetic clusters using the STRUCTURE algorithm. Assignment of individuals to genetic clusters using the STRUCTURE algorithm for K = 3 (mean LogLikelihood across three runs, -ln = 618.7), K = 4 (mean LogLikelihood across three runs, -ln = 631.7), K = 5 (mean LogLikelihood across three runs, -ln = 602.4). [file 1471-2148-11-117-S3.JPEG]

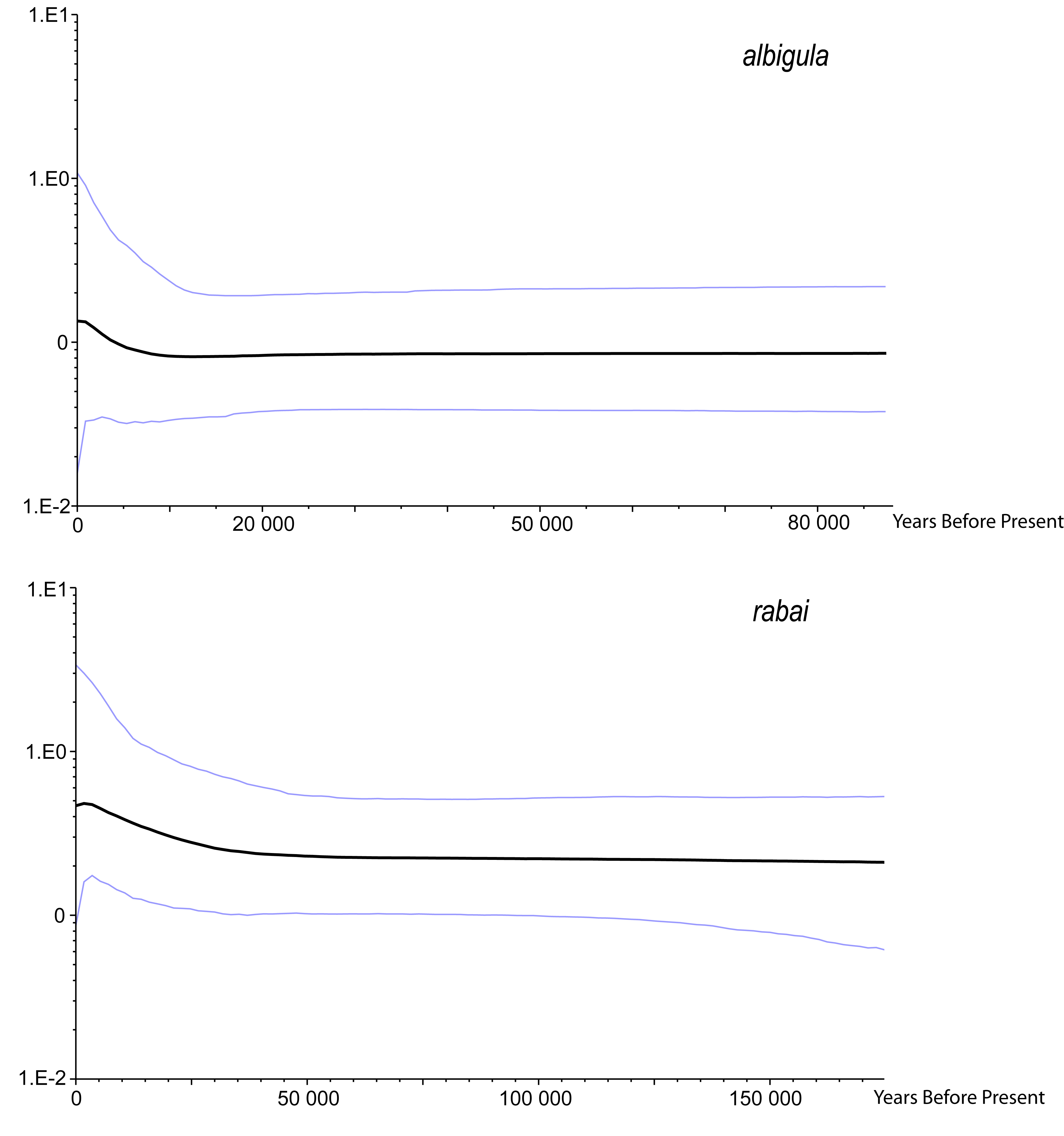

Supplement: Additional file 4 — Bayesian Skyline Plot of the mitochondrial data sets for the subspecies rabai and albigula. Bayesian Skyline Plot of the mitochondrial data sets for the subspecies rabai and albigula. [file 1471-2148-11-117-S4.JPEG]
