# Supplementary material for: Diversification across an altitudinal gradient in the Tiny Greenbul (Phyllastrephus debilis) from the Eastern Arc Mountains of Africa
Source: BMC Evol Biol. 2011 May 3;11:117. doi: 10.1186/1471-2148-11-117 (PMC3097164; doi:10.1186/1471-2148-11-117)
Supplement: Additional file 5 — List of samples used and Genbank Accession Numbers. List of samples used and Genbank Accession Numbers. [file 1471-2148-11-117-S5.DOCX]

**Additional File 5**. List of samples used and Genbank Accession Numbers. Subspecies designation according to Dickinson (2003). Acronyms: DNSM, Durban Museum of Natural History; FMNH, Field Museum of Natural History; ZMUC, Zoological Museum, University of Copenhagen. Latitude and Longitude are in decimal degrees. * Indicate samples with specimen vouchers.

| subspecies | Museum | Cat. Num | Locality | Latitude | Longitude | Country | ND2 | Fib5 | GAPDH | BRM |
| --- | --- | --- | --- | --- | --- | --- | --- | --- | --- | --- |
| *albigula* | ZMUC | 120079 * | Amani, East Usambara Mts | -5.06 | 38.63 | Tanzania | HQ716731 | HQ716840 | HQ716940 | HQ717053 |
| *albigula* | FMNH | 356725 * | Ambangulu Tea Estate, West Usambara Mts | -5.08 | 38.4 | Tanzania | HQ716817 | HQ716922 | HQ717029 | HQ717138 |
| *albigula* | FMNH | 356727 * | Ambangulu Tea Estate, West Usambara Mts | -5.08 | 38.4 | Tanzania | HQ716818 | HQ716923 | HQ717030 | HQ717139 |
| *albigula* | ZMUC | 121115 * | Lutindi, Mt. Nilo, East Usambara Mts | -4.88 | 38.63 | Tanzania | HQ716763 | HQ716872 | HQ716972 | HQ717085 |
| *albigula* | ZMUC | 121116 | Lutindi, Mt. Nilo, East Usambara Mts | -4.88 | 38.63 | Tanzania | HQ716752 | HQ716861 | HQ716961 | HQ717074 |
| *albigula* | ZMUC | 121155 | Lutindi, Mt. Nilo, East Usambara Mts | -4.88 | 38.63 | Tanzania | HQ716764 | HQ716873 | HQ716973 | HQ717086 |
| *albigula* | ZMUC | 121166 | Lutindi, Mt. Nilo, East Usambara Mts | -4.88 | 38.63 | Tanzania | HQ716761 | HQ716870 | HQ716970 | HQ717083 |
| *albigula* | ZMUC | 121173 | Lutindi, Mt. Nilo, East Usambara Mts | -4.88 | 38.63 | Tanzania | HQ716762 | HQ716871 | HQ716971 | HQ717084 |
| *albigula* | ZMUC | 122675 | Lutindi, Mt. Nilo, East Usambara Mts | -4.88 | 38.63 | Tanzania | HQ716804 | HQ716913 | HQ717012 | HQ717126 |
| *albigula* | ZMUC | 122676 | Lutindi, Mt. Nilo, East Usambara Mts | -4.88 | 38.63 | Tanzania | HQ716805 | HQ716914 | HQ717013 | HQ717127 |
| *albigula* | ZMUC | 122677 | Lutindi, Mt. Nilo, East Usambara Mts | -4.88 | 38.63 | Tanzania | HQ716798 | HQ716907 | HQ717006 | HQ717120 |
| *albigula* | ZMUC | 122682 | Lutindi, Mt. Nilo, East Usambara Mts | -4.88 | 38.63 | Tanzania | HQ716793 | HQ716902 | HQ717001 | HQ717115 |
| *albigula* | ZMUC | 129092 * | Magamba Forest, West Usambara Mts | -4.75 | 38.27 | Tanzania | HQ716784 | HQ716893 |  | HQ717106 |
| *albigula* | ZMUC | 129095 * | Magamba Forest, West Usambara Mts | -4.75 | 38.27 | Tanzania | HQ716786 | HQ716895 | HQ716994 | HQ717108 |
| *albigula* | ZMUC | 120129 | Mazumbai, West Usambara Mts | -4.77 | 38.4 | Tanzania |  |  | HQ716939 | HQ717052 |
| *albigula* | ZMUC | 120134 | Mazumbai, West Usambara Mts | -4.77 | 38.4 | Tanzania | HQ716734 | HQ716843 | HQ716943 | HQ717056 |
| *albigula* | ZMUC | 120135 * | Mazumbai, West Usambara Mts | -4.77 | 38.4 | Tanzania | HQ716735 | HQ716844 | HQ716944 | HQ717057 |
| *albigula* | ZMUC | 120148 | Mazumbai, West Usambara Mts | -4.77 | 38.4 | Tanzania | HQ716749 | HQ716858 | HQ716958 | HQ717071 |
| *albigula* | ZMUC | 120161 | Mazumbai, West Usambara Mts | -4.77 | 38.4 | Tanzania | HQ716806 | HQ716915 | HQ717014 | HQ717128 |
| *albigula* | ZMUC | 120168 * | Mazumbai, West Usambara Mts | -4.77 | 38.4 | Tanzania | HQ716789 | HQ716898 | HQ716997 | HQ717111 |
| *albigula* | ZMUC | 120185 | Mazumbai, West Usambara Mts | -4.77 | 38.4 | Tanzania | HQ716787 | HQ716896 | HQ716995 | HQ717109 |
| *albigula* | ZMUC | 120186 | Mazumbai, West Usambara Mts | -4.77 | 38.4 | Tanzania | HQ716785 | HQ716894 | HQ716993 | HQ717107 |
| *albigula* | ZMUC | 126941 | Nguru Mts., 1500 m | -6.02 | 37.55 | Tanzania | HQ716807 | HQ716916 | HQ717015 | HQ717129 |
| *albigula* | ZMUC | 126942 | Nguru Mts., 1500 m | -6.02 | 37.55 | Tanzania | HQ716797 | HQ716906 | HQ717005 | HQ717119 |
| *albigula* | ZMUC | 132848 * | Nguru South Forest Reserve, Nguru Mts | -6.05 | 37.55 | Tanzania | HQ716737 | HQ716846 | HQ716946 | HQ717059 |
| *albigula* | ZMUC | 132849 * | Nguru South Forest Reserve, Nguru Mts | -6.05 | 37.55 | Tanzania | HQ716736 | HQ716845 | HQ716945 | HQ717058 |
| *albigula* | ZMUC | 132851 | Nguru South Forest Reserve, Nguru Mts | -6.05 | 37.55 | Tanzania | HQ716739 | HQ716848 | HQ716948 | HQ717061 |
| *albigula* | ZMUC | 132860 | Nguru South Forest Reserve, Nguru Mts | -6.05 | 37.55 | Tanzania | HQ716738 | HQ716847 | HQ716947 | HQ717060 |
| *albigula* | ZMUC | 132906 * | Nguru South Forest Reserve, Nguru Mts | -6.05 | 37.55 | Tanzania | HQ716748 | HQ716857 | HQ716957 | HQ717070 |
| *albigula* | ZMUC | 132910 | Nguru South Forest Reserve, Nguru Mts | -6.05 | 37.55 | Tanzania | HQ716751 | HQ716860 | HQ716960 | HQ717073 |
| *albigula* | ZMUC | 132912 * | Nguru South Forest Reserve, Nguru Mts | -6.05 | 37.55 | Tanzania | HQ716750 | HQ716859 | HQ716959 | HQ717072 |
| *albigula* | ZMUC | 132914 | Nguru South Forest Reserve, Nguru Mts | -6.05 | 37.55 | Tanzania | HQ716745 | HQ716854 | HQ716954 | HQ717067 |
| *albigula* | ZMUC | 132928 | Nguru South Forest Reserve, Nguru Mts | -6.05 | 37.55 | Tanzania | HQ716744 | HQ716853 | HQ716953 | HQ717066 |
| *albigula* | ZMUC | 132930 | Nguru South Forest Reserve, Nguru Mts | -6.05 | 37.55 | Tanzania | HQ716747 | HQ716856 | HQ716956 | HQ717069 |
| *albigula* | ZMUC | 132934 | Nguru South Forest Reserve, Nguru Mts | -6.05 | 37.55 | Tanzania | HQ716746 | HQ716855 | HQ716955 | HQ717068 |
| *albigula* | ZMUC | 132939 | Nguru South Forest Reserve, Nguru Mts | -6.05 | 37.55 | Tanzania | HQ716727 | HQ716837 | HQ716935 | HQ717048 |
| *albigula* | ZMUC | 132940 | Nguru South Forest Reserve, Nguru Mts | -6.05 | 37.55 | Tanzania | HQ716726 | HQ716836 | HQ716934 | HQ717047 |
| *albigula* | ZMUC | 132944 | Nguru South Forest Reserve, Nguru Mts | -6.05 | 37.55 | Tanzania | HQ716729 | HQ716839 | HQ716937 | HQ717050 |
| *albigula* | ZMUC | 132947 | Nguru South Forest Reserve, Nguru Mts | -6.05 | 37.55 | Tanzania | HQ716728 | HQ716838 | HQ716936 | HQ717049 |
| *albigula* | ZMUC | 132956 | Nguru South Forest Reserve, Nguru Mts | -6.05 | 37.55 | Tanzania | HQ716723 | HQ716833 | HQ716931 | HQ717044 |
| *albigula* | ZMUC | 132973 * | Nguru South Forest Reserve, Nguru Mts | -6.05 | 37.55 | Tanzania | HQ716722 | HQ716832 | HQ716930 | HQ717043 |
| *albigula* | ZMUC | 132974 * | Nguru South Forest Reserve, Nguru Mts | -6.05 | 37.55 | Tanzania | HQ716725 | HQ716835 | HQ716933 | HQ717046 |
| *albigula* | ZMUC | 132975 * | Nguru South Forest Reserve, Nguru Mts | -6.05 | 37.55 | Tanzania | HQ716724 | HQ716834 | HQ716932 | HQ717045 |
| *albigula* | ZMUC | 132979 | Nguru South Forest Reserve, Nguru Mts | -6.05 | 37.55 | Tanzania | HQ716733 | HQ716842 | HQ716942 | HQ717055 |
| *albigula* | ZMUC | 136200 | Nguru South Forest Reserve, Nguru Mts | -6.05 | 37.55 | Tanzania | HQ716774 | HQ716883 | HQ716983 | HQ717096 |
| *albigula* | ZMUC | 136205 | Nguru South Forest Reserve, Nguru Mts | -6.05 | 37.55 | Tanzania | HQ716773 | HQ716882 | HQ716982 | HQ717095 |
| *albigula* | ZMUC | 137398 | Nguru South Forest Reserve, Nguru Mts | -6.05 | 37.55 | Tanzania | HQ716771 | HQ716880 | HQ716980 | HQ717093 |
| *albigula* | ZMUC | 137412 * | Nguru South Forest Reserve, Nguru Mts | -6.05 | 37.55 | Tanzania | HQ716782 | HQ716891 | HQ716991 | HQ717104 |
| *albigula* | ZMUC | 137446 | Nguru South Forest Reserve, Nguru Mts | -6.05 | 37.55 | Tanzania | HQ716781 | HQ716890 | HQ716990 | HQ717103 |
| *albigula* | ZMUC | 137466 | Nguru South Forest Reserve, Nguru Mts | -6.05 | 37.55 | Tanzania | HQ716783 | HQ716892 | HQ716992 | HQ717105 |
| *albigula* | ZMUC | 137469 | Nguru South Forest Reserve, Nguru Mts | -6.05 | 37.55 | Tanzania | HQ716778 | HQ716887 | HQ716987 | HQ717100 |
| *albigula* | ZMUC | 137479 * | Nguru South Forest Reserve, Nguru Mts | -6.05 | 37.55 | Tanzania | HQ716777 | HQ716886 | HQ716986 | HQ717099 |
| *albigula* | ZMUC | 137497 | Nguru South Forest Reserve, Nguru Mts | -6.05 | 37.55 | Tanzania | HQ716780 | HQ716889 | HQ716989 | HQ717102 |
| *albigula* | ZMUC | 137501 | Nguru South Forest Reserve, Nguru Mts | -6.05 | 37.55 | Tanzania | HQ716779 | HQ716888 | HQ716988 | HQ717101 |
| *albigula* | ZMUC | 137508 | Nguru South Forest Reserve, Nguru Mts | -6.05 | 37.55 | Tanzania | HQ716758 | HQ716867 | HQ716967 | HQ717080 |
| *albigula* | ZMUC | 137509 * | Nguru South Forest Reserve, Nguru Mts | -6.05 | 37.55 | Tanzania | HQ716757 | HQ716866 | HQ716966 | HQ717079 |
| *albigula* | ZMUC | 137510 * | Nguru South Forest Reserve, Nguru Mts | -6.05 | 37.55 | Tanzania | HQ716760 | HQ716869 | HQ716969 | HQ717082 |
| *albigula* | ZMUC | 137522 | Nguru South Forest Reserve, Nguru Mts | -6.05 | 37.55 | Tanzania | HQ716759 | HQ716868 | HQ716968 | HQ717081 |
| *albigula* | ZMUC | 137526 | Nguru South Forest Reserve, Nguru Mts | -6.05 | 37.55 | Tanzania | HQ716754 | HQ716863 | HQ716963 | HQ717076 |
| *albigula* | ZMUC | 137531 | Nguru South Forest Reserve. Nguru Mts | -6.05 | 37.55 | Tanzania | HQ716753 | HQ716862 | HQ716962 | HQ717075 |
| *debilis* | DNSM | 23510 * | Dondo, Beira | -19.82 | 34.87 | Mozambique | HQ716808 |  |  |  |
| *debilis* | DNSM | 23514 * | Dondo, Beira | -19.82 | 34.87 | Mozambique | HQ716813 | HQ716919 | HQ717025 |  |
| *debilis* | DNSM | 23521 * | Dondo, Beira | -19.82 | 34.87 | Mozambique | HQ716816 |  | HQ717027 |  |
| *debilis* | DNSM | 23524 * | Dondo, Beira | -19.82 | 34.87 | Mozambique |  |  | HQ717023 | HQ717136 |
| *debilis* | DNSM | 23527 * | Dondo, Beira | -19.82 | 34.87 | Mozambique |  |  | HQ717028 | HQ717137 |
| *debilis* | DNSM | 23528 * | Dondo, Beira | -19.82 | 34.87 | Mozambique |  |  | HQ717016 | HQ717130 |
| *debilis* | DNSM | 23505 * | Inhamane | -23.87 | 35.38 | Mozambique |  |  | HQ717021 | HQ717134 |
| *debilis* | DNSM | 25064 * | Lagoa Ura | -19.71 | 34.33 | Mozambique |  |  |  | HQ717132 |
| *debilis* | ZMUC | 74473 * | Lindi | -10 | 39.72 | Tanzania | HQ716827 |  | HQ717037 |  |
| *debilis* | ZMUC | 119477 * | Litipo Forest | -10.03 | 39.48 | Tanzania | HQ716772 | HQ716881 | HQ716981 | HQ717094 |
| *debilis* | ZMUC | 67319 * | Lrisite River Forest, Vimba | -19.92 | 32.72 | Zimbabwe | HQ716829 |  | HQ717039 |  |
| *debilis* | DNSM | 20147 * | Mapinhane | -22.27 | 35.12 | Mozambique | HQ716810 |  | HQ717018 | HQ717131 |
| *debilis* | DNSM | 20146 * | Massinga | -22.92 | 35.3 | Mozambique |  | HQ716917 | HQ717019 | HQ717133 |
| *debilis* | ZMUC | 119971 | Pindiro Forest | -9.5 | 39.23 | Tanzania | HQ716775 | HQ716884 | HQ716984 | HQ717097 |
| *debilis* | DNSM | 29193 * | Vimba | -19.92 | 32.72 | Zimbabwe | HQ716811 | HQ716918 | HQ717022 | HQ717135 |
| *debilis* | DNSM | 29194 * | Vimba | -19.92 | 32.72 | Zimbabwe | HQ716815 | HQ716921 | HQ717026 |  |
| *debilis* | DNSM | 29195 * | Vimba | -19.92 | 32.72 | Zimbabwe |  |  | HQ717020 |  |
| *debilis* | DNSM | 29196 * | Vimba | -19.92 | 32.72 | Zimbabwe | HQ716812 |  | HQ717024 |  |
| *debilis* | DNSM | 29198 * | Vimba | -19.92 | 32.72 | Zimbabwe | HQ716809 |  | HQ717017 |  |
| *debilis* | DNSM | 29199 * | Vimba | -19.92 | 32.72 | Zimbabwe | HQ716814 | HQ716920 |  |  |
| *debilis* | DNSM | 29200 * | Vimba | -19.92 | 32.72 | Zimbabwe |  |  |  |  |
| *rabai* | ZMUC | 122584 | Bombo East, East Usambara Mts | -4.83 | 38.72 | Tanzania | HQ716770 | HQ716879 | HQ716979 | HQ717092 |
| *rabai* | ZMUC | 129599 | Chamanyani Mvuha Forest Reserve | -7.18 | 37.82 | Tanzania | HQ716790 | HQ716899 | HQ716998 | HQ717112 |
| *rabai* | ZMUC | 129600 | Chamanyani Mvuha Forest Reserve | -7.18 | 37.82 | Tanzania | HQ716791 | HQ716900 | HQ716999 | HQ717113 |
| *rabai* | ZMUC | 121105 * | Dondwe Forest | -5.93 | 38.07 | Tanzania | HQ716766 | HQ716875 | HQ716975 | HQ717088 |
| *rabai* | ZMUC | 121106 * | Dondwe Forest | -5.93 | 38.07 | Tanzania | HQ716767 | HQ716876 | HQ716976 | HQ717089 |
| *rabai* | ZMUC | 120023 * | Kambai Forest, East Usambara Mts | -4.98 | 38.77 | Tanzania | HQ716776 | HQ716885 | HQ716985 | HQ717098 |
| *rabai* | ZMUC | 120028 | Kambai Forest, East Usambara Mts | -4.98 | 38.77 | Tanzania | HQ716721 | HQ716831 | HQ716929 | HQ717042 |
| *rabai* | ZMUC | 132678 | Mt. Kanga Forest Reserve | -5.98 | 37.7 | Tanzania | HQ716800 | HQ716909 | HQ717008 | HQ717122 |
| *rabai* | ZMUC | 132688 | Mt. Kanga Forest Reserve | -5.98 | 37.7 | Tanzania | HQ716803 | HQ716912 | HQ717011 | HQ717125 |
| *rabai* | ZMUC | 132701 | Mt. Kanga Forest Reserve | -5.98 | 37.7 | Tanzania | HQ716802 | HQ716911 | HQ717010 | HQ717124 |
| *rabai* | ZMUC | 132702 | Mt. Kanga Forest Reserve | -5.98 | 37.7 | Tanzania | HQ716741 | HQ716850 | HQ716950 | HQ717063 |
| *rabai* | ZMUC | 132703 | Mt. Kanga Forest Reserve | -5.98 | 37.7 | Tanzania | HQ716740 | HQ716849 | HQ716949 | HQ717062 |
| *rabai* | ZMUC | 132719 | Mt. Kanga Forest Reserve | -5.98 | 37.7 | Tanzania | HQ716822 | HQ716927 | HQ717034 | HQ717143 |
| *rabai* | ZMUC | 132720 * | Mt. Kanga Forest Reserve | -5.98 | 37.7 | Tanzania | HQ716743 | HQ716852 | HQ716952 | HQ717065 |
| *rabai* | ZMUC | 132724 * | Mt. Kanga Forest Reserve | -5.98 | 37.7 | Tanzania | HQ716742 | HQ716851 | HQ716951 | HQ717064 |
| *rabai* | ZMUC | 134305 | Mt. Kanga Forest Reserve | -5.98 | 37.7 | Tanzania | HQ716730 |  | HQ716938 | HQ717051 |
| *rabai* | ZMUC | 134311 | Mt. Kanga Forest Reserve | -5.98 | 37.7 | Tanzania | HQ716732 | HQ716841 | HQ716941 | HQ717054 |
| *rabai* | ZMUC | 119268 | Mt. Kanga Forest Reserve | -5.98 | 37.7 | Tanzania | HQ716755 | HQ716864 | HQ716964 | HQ717077 |
| *rabai* | ZMUC | 119269 | Mt. Kanga Forest Reserve | -5.98 | 37.7 | Tanzania | HQ716756 | HQ716865 | HQ716965 | HQ717078 |
| *rabai* | ZMUC | 92265 * | Kidugallo | -6.78 | 38.2 | Tanzania | HQ716825 |  | HQ717040 |  |
| *rabai* | ZMUC | 92264 * | Kingolwira | -6.77 | 37.39 | Tanzania | HQ716830 |  | HQ717041 |  |
| *rabai* | ZMUC | 92266 * | Mogana, Uluguru Mts | -7.05 | 37.67 | Tanzania | HQ716826 |  | HQ717036 |  |
| *rabai* | ZMUC | 122707 * | Nguu Mts. | -5.47 | 37.48 | Tanzania | HQ716792 | HQ716901 | HQ717000 | HQ717114 |
| *rabai* | ZMUC | 121048 * | Nkubege Coastal Forest | -6.95 | 39.17 | Tanzania | HQ716788 | HQ716897 | HQ716996 | HQ717110 |
| *rabai* | ZMUC | 121051 * | Nkubege Coastal Forest | -6.95 | 39.17 | Tanzania | HQ716799 | HQ716908 | HQ717007 | HQ717121 |
| *rabai* | ZMUC | 121056 | Nkubege Coastal Forest | -6.95 | 39.17 | Tanzania | HQ716794 | HQ716903 | HQ717002 | HQ717116 |
| *rabai* | ZMUC | 121068 | Nkubege Coastal Forest | -6.95 | 39.17 | Tanzania | HQ716796 | HQ716905 | HQ717004 | HQ717118 |
| *rabai* | ZMUC | 121069 | Nkubege Coastal Forest | -6.95 | 39.17 | Tanzania | HQ716795 | HQ716904 | HQ717003 | HQ717117 |
| *rabai* | ZMUC | 121087 | Nkubege Coastal Forest | -6.95 | 39.17 | Tanzania | HQ716801 | HQ716910 | HQ717009 | HQ717123 |
| *rabai* | ZMUC | 141034 * | Pugu Hills | -6.88 | 39.08 | Tanzania | HQ716819 | HQ716924 | HQ717031 | HQ717140 |
| *rabai* | ZMUC | 141039 * | Pugu Hills | -6.88 | 39.08 | Tanzania | HQ716824 |  |  | HQ717145 |
| *rabai* | ZMUC | 141062 * | Pugu Hills | -6.88 | 39.08 | Tanzania | HQ716820 | HQ716925 | HQ717032 | HQ717141 |
| *rabai* | ZMUC | 141063 | Pugu Hills | -6.88 | 39.08 | Tanzania | HQ716821 | HQ716926 | HQ717033 | HQ717142 |
| *rabai* | ZMUC | 141067 | Pugu Hills | -6.88 | 39.08 | Tanzania | HQ716823 | HQ716928 | HQ717035 | HQ717144 |
| *rabai* | ZMUC | 117617 | Pugu Hills | -6.88 | 39.08 | Tanzania | HQ716768 | HQ716877 | HQ716977 | HQ717090 |
| *rabai* | ZMUC | 117618 | Pugu Hills | -6.88 | 39.08 | Tanzania | HQ716765 | HQ716874 | HQ716974 | HQ717087 |
| *rabai* | ZMUC | 119510 | Sokoke Forest | -3.48 | 39.83 | Kenya | HQ716769 | HQ716878 | HQ716978 | HQ717091 |
| *rabai* | ZMUC | 92267 * | Uluguru Mts | -7.05 | 37.67 | Tanzania | HQ716828 |  | HQ717038 |  |
