# Supplementary material for: Diversification across an altitudinal gradient in the Tiny Greenbul (Phyllastrephus debilis) from the Eastern Arc Mountains of Africa
Source: BMC Evol Biol. 2011 May 3;11:117. doi: 10.1186/1471-2148-11-117 (PMC3097164; doi:10.1186/1471-2148-11-117)
Supplement: Additional file 6 — List of loci sequenced and primers used for PCR amplification and sequencing. List of loci sequenced and primers used for PCR amplification and sequencing. [file 1471-2148-11-117-S6.DOCX]

**Additional File 6**. List of loci sequenced and primers used for PCR amplification and sequencing. The –*deb*- acronyms refer to internal primers defined to amplify and sequence the museum specimens.

| Locus | Location on the chicken genome | primers sequence (5’-3’) | References |
| --- | --- | --- | --- |
| GAPDH intron-11 | Chromosome 1 | G3P14b: AAGTCCACAACACGGTTGCTGTA, G3PintL1: GAACGACCATTTTGTCAAGCTGGTT, G3P13:TCCACCTTTGATGCGGGTGCTGGCAT  *deb*GPintR: ACAACTGAACTCCCATCTACC  *deb*GPintF : GTTACTGCTGGTGATCCAGG | Fjeldså et al. (2003), This study |
| FGB intron-5 | Chromosome 4 | Fib5: CGCCATACAGAG TATACTGTGACAT, Fib6: GCCATCCTGGCGATTCTGAA  *deb*FibintR : GTAAGAAATGAGCCAGGCTGTC  *deb*FibintF : CAGAATAGGATACTTCTGTAGTG | Fuchs et al. (2004), This study |
| BRM intron-15 | Chromosome Z | BRM15F: AGCACCTTTGAACAGTGGTT, BRM15R: TACTTTATGGAGACGACGGA | Goodwin (1997) |
